# High Expression of CEMIP Correlates Poor Prognosis and the Tumur Microenvironment in Breast Cancer as a Promisingly Prognostic Biomarker

**DOI:** 10.3389/fgene.2021.768140

**Published:** 2021-12-13

**Authors:** Xingxing Dong, Yalong Yang, Qianqian Yuan, Jinxuan Hou, Gaosong Wu

**Affiliations:** Department of Thyroid and Breast Surgery, Zhongnan Hospital of Wuhan University, Wuhan, China

**Keywords:** breast cancer, CEMIP, prognosis, tumor microenvironment, biomarker

## Abstract

Cell migration-inducing hyaluronidase 1 (CEMIP), a Wnt-related protein and also known as KIAA1199, is implicated in the process of metastatic colonization in a variety of malignant tumors, including breast cancer (BC), which is one of the most frequently diagnosed tumors in women worldwide. In this study, multiple public databases, online analytical tools, and bioinformatics approaches were applied to explore the expression levels, regulatory mechanisms, and biological functions of CEMIP in BC. We illustrated that *CEMIP* was highly expressed in various kinds of carcinomas, including BC, especially advanced subtypes, and predicted less favorable prognosis (negatively associated with overall survival) in BC patients, which might be an independent prognostic factor. Then, we revealed that the mutation and high expression of *CEMIP* might lead to it as an oncogene. We also demonstrated that *TP53* mutation, DNA hypo-methylation, and the expression changes of three potential upstream transcription factors (*EZH2, EGR1*, and *JUN*) of *CEMIP* were likely to cause the hyperexpression of *CEMIP* in BC*.* Moreover, our findings suggested that CEMIP might exert its carcinogenic roles in the tumor microenvironment via participation in the extracellular matrix formation, increasing cancer-associated fibroblast (CAF), M2 macrophage, and neutrophil infiltration and decreasing CD8^+^ T cell infiltration. In summary, our study provided more solid evidence for CEMIP as a prognostic and metastatic biomarker and a potential therapeutic target in BC. Of course, these findings also need more confirmations of basic experiments and further clinical trials in the future.

## Introduction

Cancer of the female breast is the leading malignancy worldwide with the highest incidence of 11.7% and the fourth in mortality (Sung et al., 2021). Some known risk factors contribute to the development of breast cancer (BC), for instance, gene mutations and lactation deficiency (Harbeck et al., 2019). The genetic predisposition of *BRCA1* and *BRCA2* mutations inherited from the family has been widely accepted. For sporadic BC, the advanced maternal age for a first pregnancy, early menarche, lack of breastfeeding, and late-onset menopause are recognized as risk factors. Besides, some modifiable risk factors, like obesity, physical inactivity, and alcohol use should also be noted (Harbeck et al., 2019). However, the exact mechanism of BC is still ambiguous. BC is highly heterogeneous (Aleskandarany et al., 2018) up to now, which can be divided into four major subtypes according to molecular biomarkers (ER/PR, HER2, and Ki^−^67) in clinical. In detail, they are luminal A (ER^+^ and/or PR^+^, HER2^−^, Ki-67^−^), luminal B (ER^+^ and/or PR^+^, HER2^+^, Ki-67^+^), HER2^+^ (ER^−^ and PR^−^, HER2^+^), and basal or triple-negative (ER^−^, PR^−^, and HER2^−^) (Dai et al., 2015). The clinical treatment and prognosis of BC patients vary depending on these subtypes. Based on the worldwide clinical retrospective analysis, patients with luminal BC have the best outcomes with surgery, endocrine therapy, and chemotherapy; the HER2^+^ second and the patients with triple-negative BC (TNBC) usually have the worst prognosis due to limited therapeutic options (Waks and Winer, 2019). Of course, other prognostic factors like age, stage, tumor grade, and lymphovascular status should be taken into consideration as well (Harbeck et al., 2019). Fortunately, the PD-L1 inhibition as a single-molecule target for therapy has been proved to ameliorate the progression-free survival (PFS) in TNBC patients, and CDK4/6 inhibitors substantially improve PFS of BC patients with endocrine resistance in recent years, although not for all BC patients (Schmid et al., 2018; Turner et al., 2018; Harbeck et al., 2019). Thereby, more effective therapeutic targets are to be explored urgently.

In recent decades, with the rise of high-throughput technologies and next-generation sequencing (NGS), followed by bioinformatics approaches and a variety of online analysis tools applied, the search for the key targeted genes in tumor genesis and progress has become a trend in tumor research in an attempt to reveal the mechanism of tumor genesis and development and thus to provide more precise treatment for patients and improve their prognosis. For instance, by applying these biological analysis tools, Lou et al. (2021) elucidated that *SEMA3F* was associated with poor prognosis and tumor immune infiltration of hepatocellular carcinoma, mediated by the TMPO-AS1/SNHG16-let-7c-5p axis. Cui et al. (2020) carried out a comprehensive pan-cancer analysis of the oncogenic role of *SND1* in human tumors, and Zeng et al. (2019) evaluated the potential of CXC chemokines as therapeutic targets and prognostic biomarkers in renal cell carcinoma.

Cell migration-inducing protein (*CEMIP*), previously known as *KIAA1199* or hyaluronan binding protein (*HYBID*), included in the Human Unidentified Gene-Encoded (HUGE) large protein database, is located on human chromosome 15q25.1 that encodes a 153 kDa protein which contains two GG domains and a special G8 domain (Li et al., 2017; Liu et al., 2021a). CEMIP is a kind of secreted protein, identified as an inner ear-specific protein at first, and mutations in it are related to non-syndromic hearing loss. In the past decades, an increasing number of studies have revealed that high expression of *CEMIP* promotes numerous malignancy progresses and metastasis and predicts poor prognosis of cancer patients, including breast (Jami et al., 2014), colorectal (Fink et al., 2015), liver, gastric, pancreatic, lung (Li et al., 2020a), prostate, cholangitis (Zhai et al., 2020), ovarian (Shen et al., 2019), and papillary thyroid cancers (Liu et al., 2021a). On the other hand, accumulated cell- and animal-based evidence shows that the over-expression of *CEMIP* could enhance proliferation, survival (Michishita et al., 2006), adhesion, motility, invasiveness, and epithelial-to-mesenchymal transition (EMT) (Liu et al., 2021b) of various cancer cells. Some researchers have elucidated that CEMIP is involved in Wnt/β-catenin, MEK/ERK, and PI3K/Akt signal pathways that contribute to the tumorigenesis (Liu et al., 2021a). A recent study reported that CEMIP can accelerate BC cell proliferation and migration by activating the STAT3 pathway (Chen et al., 2021). It is widely acknowledged that the expression of *CEMIP* is regulated by both genetic and epigenetic mechanisms. The two key transcription factors are nuclear factor-κB (NF-κB) (Shostak et al., 2014) and activator protein-1 (AP-1), although the basic promoter activity of this gene mainly lied in the DNA methylation of the CpG island (Kuscu et al., 2012). For example, *CEMIP* over-expression was associated with hypomethylation of the CpG island in BC (Kuscu et al., 2012). In addition, the increased presence of lysine 4 of histone H3 trimethylation (H3K4me3) was reported to be an activation marker for *CEMIP* transcription, and reduced H3K27me3 was demonstrated to promote its expression in the development of BC (Hsieh et al., 2020; Liu et al., 2021a). Recently, several microRNAs and pro-inflammatory cytokines have been described to participate in the expression regulation of *CEMIP* as concluded in a review (Liu et al., 2021a). All works above indicate that *CEMIP* plays an important role in the oncogenesis and progress of carcinomas, and the exact mechanism remains to be explored. Moreover, the correlations of *CEMIP* with tumor immune infiltration in BC are still not determined.

In the present study, we performed expression analysis for *CEMIP* in different kinds of human cancers, particularly in BC, and assessed the association of *CEMIP* expression with the prognosis of patients with BC. Next, the potentially genetic regulatory mechanisms of *CEMIP*, including DNA methylation, genetic alteration, and upstream transcription factors, were explored in BC and other cancers. Then, we also manipulated functional annotation of *CEMIP*-related genes and *CEMIP*-interacted kinases and determined the correlation of *CEMIP* mRNA expression with the infiltration level of immune cells, biomarkers of immune cells, and immune checkpoints in BC. Finally, gene set enrichment analysis (GSEA) of a single gene was performed to identify the underlying pathways and hallmark perturbations caused by *CEMIP* in BC. All analyses were principally based on TCGA and GEO databases. In conclusion, our findings uncover that the up-regulation of *CEMIP* mediated by *TP53* mutation, DNA hypomethylation, and transcription factors correlates with worse outcomes and higher immune cell infiltration levels of patients with BC.

## Materials and Methods

### mRNA and Protein Expression of *CEMIP* in Normal Tissues and Cellular Localization Analysis

First, GeneCards®: The Human Gene Database (https://www.genecards.org/) was used to explore mRNA and protein expression of *CEMIP* in normal tissues and cells in the “expression module” and visualize its subcellular locations in the “Localization module”. The Human Protein Atlas (https://www.proteinatlas.org/) was as well applied to visualize the location of CEMIP.

### *CEMIP* Expression Analysis Among Tumors

Subsequently, the “Gene_DE Module” in the TIMER2.0 (Li et al., 2020b) (tumor immune estimation resource, version 2) webserver (http://timer.cistrome.org/) was applied to estimate the *CEMIP* mRNA expression level in all The Cancer Genome Atlas (TCGA) (Tomczak et al., 2015) cancer types compared to their corresponding adjacent normal tissues, which were displayed by box plots. Next, we employed the UALCAN (Chandrashekar et al., 2017) (http://ualcan.path.uab.edu/analysis-prot.html) web resource to investigate *CEMIP* expression among 1097 BC samples and 114 normal samples of TCGA data based on sample types (that is., BC versus normal tissues), individual cancer stages, patients’ ages, major BC subclasses, nodal metastasis statuses, and *TP53* mutation statuses with the “Expression Link” of the “TCGA analysis module”. Then, Breast Cancer Gene-Expression Miner v4.7 (Jézéquel et al., 2021) (bc-GenExMiner v4.7, http://bcgenex.ico.unicancer.fr/BC-GEM/GEM-Accueil.php?js=1) was applied to validate *CEMIP* expression with DNA microarray data (*n* = 11,359) in the “EXPRESSION” of the “ANALYSIS Module” based on intrinsic molecular subtypes (PAM50 subtypes), patients’ ages, nodal metastasis statuses, and *TP53* mutation statuses. Furthermore, we also downloaded the GSE42568 dataset (raw CEL file and GPL file) from The Gene Expression Omnibus (Edgar et al., 2002) (GEO, https://www.ncbi.nlm.nih.gov/geo/), which was based on GPL570 Platforms ([HG-U133_Plus_2] Affymetrix Human Genome U133 Plus 2.0 Array) and contained 104 primary BC and 17 normal breast biopsies gene chips. After quality control, a total of 94 BC and 14 normal breast samples were obtained, based on which we identified the differentially expressed genes (DEGs) in BC compared with normal samples using R package limma (Smyth, 2005) with log_2_Fold Change > 1, adjust *p*-value < 0.05 for expression validation of *CEMIP* and the following analysis.

### Survival Analysis

We first performed survival analysis through the Kaplan-Meier plotter (Lanczky et al., 2010) (https://kmplot.com/analysis/index.php?p=service) based on the GEO database and divided the samples into high-expression and low-expression cohorts according to the median expression value of *KIAA1199* (one of alias of *CEMIP*) for exploring the associations of its expression level with patients overall, relapse-free, and distant metastasis-free survival (OS, RFS, and DMFS). Next, Breast Cancer Gene-Expression Miner v4.7 (Campone et al., 2012) as well was applied to carry out survival analysis based on DNA microarrays (*n* = 11,359) in “PROGNOSIS” of the “ANALYSIS Module”, with which we explored the association of *CEMIP* expression level with patients’ OS, disease-free survival (DFS), and DMFS among all BC patients, ER/PR-positive patients, and ER/PR-negative patients. Moreover, we downloaded the TCGA dataset of BC, including gene expression RNA-seq data (*n* = 1,218), clinical phenotype data (*n* = 1,247) data, curated survival data (*n* = 1,236), and somatic mutation data (MC3 gene-level non-silent mutation, *n* = 791) from the UCSC Xena web (https://xenabrowser.net/DATAPAGES/) for the univariate and multivariate OS analysis using the Cox Proportional Hazards model carried out by R package survival. We first applied separate univariate Cox regressions to assess the statistical significance for each of the variables with OS, comprising BC patient stage, age, ER/PR/HER2 status, molecular subtype, tumor/node/metastasis (TNM) status, *TP53* mutation status, and the expression level of *CEMIP*, and then performed multivariate Cox regression analysis with these variables.

### Promoter Methylation and Genetic Alteration Analysis

Promoter methylation analysis was as well implemented using the UALCAN web based on TCGA data containing 793 BC samples and 97 normal samples, of which the BC samples were divided into different groups according to individual cancer stages, patient age, and major BC subclasses. The genetic alteration analysis of *CEMIP* was carried out in the cBioPortal (Cerami et al., 2012) web (https://www.cbioportal.org/) following these steps: (1) select “Breast”; (2) choose 11 studies (consisting of 4717 samples) except for “TCGA, Cell 2015”, “TCGA, Nature 2012”, and “TCGA, PanCancer Atlas” in the “Invasive Breast Carcinoma” section; (3) click “Query By Gene”; (4) input “CEMIP”; (5) submit Query; and (6) choose “Cancer Type Detailed” in “Cancer Types Summary” to obtain the genomic alteration of CEMIP and choose the “Mutations” option to acquire diagrams of mutation sites.

### Upstream Transcription Factor and Kinase Interaction Analysis

ARCHS^4^ (Lachmann et al., 2018) (https://maayanlab.cloud/archs4/help.html) was applied to predict the upstream transcription factor (TF) targets and kinase interactions of *CEMIP* in humans, which predicted upstream TFs based on ChIP-seq data from the ChEA and ENCODE gene set libraries and predicted protein kinases based on known kinase substrates from KEA. Subsequently, we employed the cBioPortal web to estimate genetic alterations of predicted TFs; used the Draw Venn Diagram online tool (http://bioinformatics.psb.ugent.be/webtools/Venn/) to obtain the differentially expressed upstream TF and kinase targets of *CEMIP* in BC by taking the intersection of all predicted upstream TFs, kinase targets of *CEMIP*, and DEGs identified from the GSE42568 dataset; and further validated their expression levels by the UALCAN web. For validated TFs, Breast Cancer Gene-Expression Miner v4.7 (Jezequel et al., 20132013) was applied to explore the correlations of their expression levels with *CEMIP* and investigate the correlations of significantly correlated TFs with the survival of BC patients, while the JASPAR^2022^ database (https://jaspar.genereg.net/) was used to predict binding sites of transcription factors in the *CEMIP* promoter region. Meanwhile, the functional annotation of validated kinase targets and *CEMIP* was investigated by using the Metascape (Zhou et al., 2019) resource (http://metascape.org).

### *CEMIP* Interacted and Correlated Gene Analysis

We first acquired *CEMIP*-interacted genes through the STRING (Szklarczyk et al., 2019) website (https://string-db.org/) with default settings, and then, we put “CEMIP” into the UALCAN web in “TCGA” gene analysis with breast invasive cancer of the TCGA dataset to explore *CEMIP*-correlated genes in the “Correlation” module. Next, we again employed the STRING tool to construct the protein–protein interaction (PPI) network of *CEMIP*-interacted genes and *CEMIP*-correlated genes with Pearson correlation coefficient ≥ 0.4 and reproduced the network using Cytoscape (Smoot et al., 2011). Moreover, we also performed the Gene Ontology (GO) (Ashburner et al., 2000) function and Kyoto Encyclopedia of Genes and Genomes (KEGG) (Kanehisa et al., 2019) pathway enrichment analysis of these genes with R package clusterProfiler (version 3.16.1).

### Immune Infiltration Analysis

We also used TIMER2.0 to evaluate the correlations between the expression level of *CEMIP* and the infiltration levels of immune cells, including CD8^+^ T cells, CD4^+^ T cells, B cells, macrophages, neutrophils, dendritic cells (DCs), natural killer (NK) cells, and CAF in the “Gene module” of the “Immune Association” section with all algorithms provided, like EPIC, TIMER, CIBERSORT, CIBERSORT-ABS, QUANTISEQ, XCELL, and MCPCOUNTER algorithms. Then, for obviously correlated immune cells, we further employed the Breast Cancer Gene-Expression Miner v4.7 tool to estimate the correlations between the expression of *CEMIP* and the biomarkers of immune cells as well as three well-known immune checkpoints (PD1, PD-L1, and CTLA-4).

### Gene Set Enrichment Analysis of *CEMIP*

Gene set enrichment analysis (GSEA) (Subramanian et al., 2005) is a powerful method to annotate gene expression data based on defined gene sets consisting of genes that have been proved to have common biological functions, chromosomal location or regulation, gene expression data, and information of phenotypes of samples. Herein, we used GSEA software (version 4.1.0) to assess the pathway variations correlated with the expression level of *CEMIP* based on the GSE42568 dataset by dividing BC samples into high/low-expression groups of *CEMIP* according to its median expression value with the cutoff: nominal *p*-value < 0.05.

### Statistical Analysis

In this study, the statistical analysis carried out by online tools was automatically calculated and the *p*-value, log rank *p*-value, or nominal *p*-value < 0.05 was considered as statistically significant. With the analysis performed by R, adjust *p*-value and *p*-value < 0.05 were considered as statistically significant.

## Results

### The mRNA and Protein Expression of *CEMIP* in Normal Tissues, Immune Cells, and Cellular Localization

We explored the expression profiling of *CEMIP* across different normal tissues, immune cells, and its cellular localization through two public protein databases. As displayed in Supplementary Figure S1, the mRNA of *CEMIP* is detected in most tissues using RNA-seq and microarray approaches, such as whole blood, the brain, lungs, the pancreas, the skin, etc., showing low tissue specificity, while in the breast, it can only be probed with RNA-seq (Supplementary Figure S1A). Moreover, *CEMIP* is expressed in immune cells, especially in T-reg cells, plasmacytoid DCs, naive B cells, memory B cells, and naive CD8^+^ T cells (Supplementary Figure S1B). At the protein level, *CEMIP* was distinctly detected in plasma, pancreas, and bone marrow stromal cells and stem cells, while in the breast, it was rarely expressed (Supplementary Figure S1C). On the other hand, we also dug into cellular localization of *CEMIP*, which was discovered in the plasma membrane, extracellular regions, nucleus, and endoplasmic reticulum with confidence = 5 according to GeneCards^®^: The Human Gene Database, while in The Human Protein Atlas, CEMIP was predicted to be secreted (Supplementary Figure S1D).

### The Expression of *CEMIP* in Cancers

**FIGURE 1:**
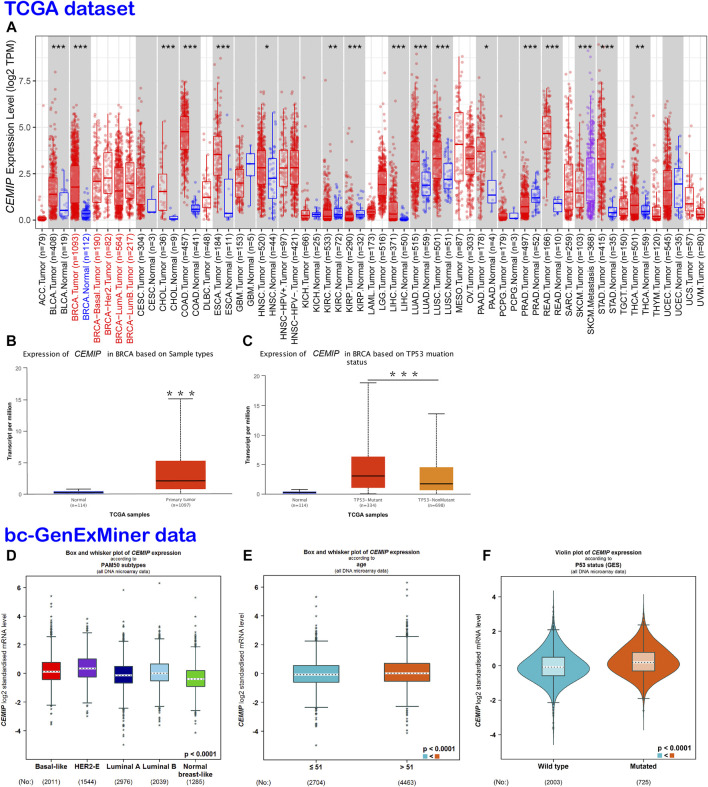
Expression analysis of *CEMIP* across a variety of cancers and clinical features of BC. **(A)** Expression profile of *CEMIP* in various cancers analyzed by TIMER2.0 based on the TCGA database. *p < 0.05; **p < 0.01; ***p < 0.001. **(B,C)** Expression analysis of *CEMIP* performed by the UALCAN web based on sample types and *TP53* mutation status with the TCGA database. ***p < 0.001. **(D–F)** Expression analysis of *CEMIP* carried out through the Breast Cancer Gene-Expression Miner v4.7 resource based on BC subtype, patient age, and *TP53* mutation status with 11,359 DNA microarrays of bc-GenExMiner data. bc-GenExMiner, the Breast Cancer Gene-Expression Miner; TCGA, The Cancer Genome Atlas.

TIMER2.0 was employed to investigate the mRNA expression level of *CEMIP* across a variety of cancers based on the TCGA dataset, which was expressed higher in 13 kinds of cancers other than normal controls (*p* < 0.05), including BC, but lower in other three types of cancer (Figure 1A). With UALCAN web tools, we again observed increased expression of *CEMIP* in BC relative to normal samples with statistical significance (*p* < 0.001) (Figure 1B) and further analyzed the expression level of *CEMIP* in BC based on subclasses (luminal, HER2^+^, and TNBC), individual cancer stages, patients’ age, nodal metastasis status, and *TP53* mutation status between 114 normal and 1,097 primary BC of TCGA. However, no significant difference existed among these clinical features (Supplementary Figures S2A–D) except that *CEMIP* was significantly expressed higher in the *TP53*-mutant group compared to the *TP53*-non-mutant group (Figure 1C). Applying the Breast Cancer Gene-Expression Miner v4.7 resource, which integrated almost all BC data comprising DNA microarrays (*n* = 11,359, most of which were obtained from the GEO dataset) and RNA-seq (*n* = 4712, TCGA data), we validated the over-expression of *CEMIP* in BC and the *TP53*-mutant group (Figures 1D,F) and found higher expression of *CEMIP* in basal-like/TNBC and HER2^+^ BC compared with luminal BC (Figure 1D). Meanwhile, the expression of *CEMIP* showed discrepancy based on BC patients’ age as well, higher in patients whose age was over 51 years, but without significant difference based on nodal metastasis status (Figure 1E; Supplementary Figure S2E). In addition, using bioinformatics approaches, we identified a total of 3,869 DEGs based on the GSE42568 dataset and also found that *CEMIP* expression was higher in BC than normal breast samples (log_2_fold Change = 1.20, adjust *p*-value < 0.0001).

### The Prognostic Value of *CEMIP* in Patients With BC

**FIGURE 2:**
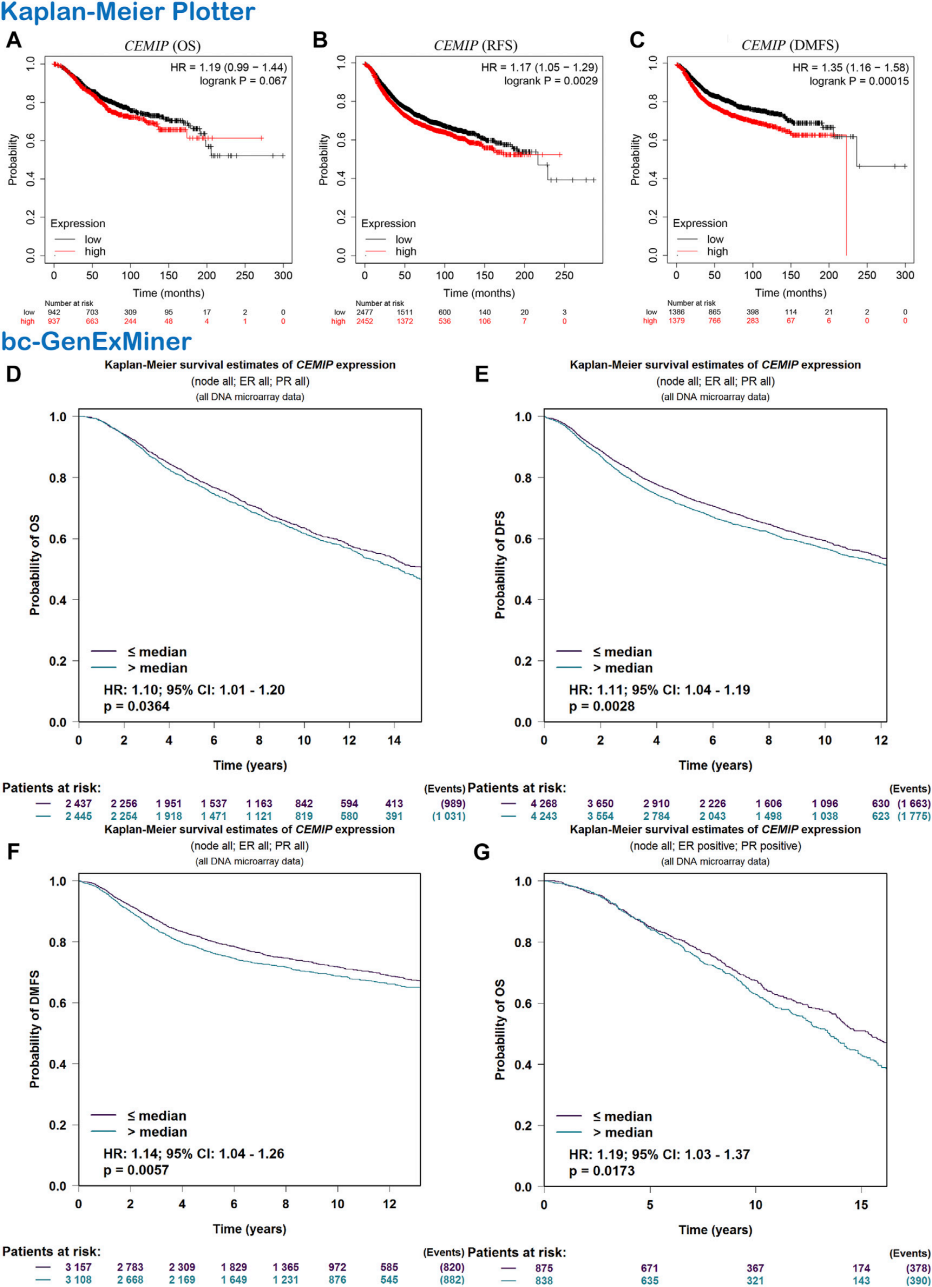
Survival analysis. **(A–C)** OS, RFS, and DMFS plots of *CEMIP* in BC with the Kaplan-Meier plotter. **(D–F)** OS, DFS, and DMFS plots of *CEMIP* in all BC subtypes with the bc-GenExMiner. **(G)** OS plot of *CEMIP* in ER/PR^+^ BC with bc-GenExMiner. Log rank *p* < 0.05 and *p* < 0.05 were considered to be statistically significant. OS, overall survival; RFS, elapse-free survival; DMFS, distant metastasis-free survival; DFS, disease-free survival.

Subsequently, we evaluated the correlation between *CEMIP* expression level and BC patients’ outcomes. The Kaplan-Meier plotter was first applied to carry out survival analysis, according to which no significant correlation was displayed between patients’ OS and the expression level of *CEMIP* (Figure 2A, *p* > 0.05), while significantly negative associations were observed between patients’ RFS and DMFS with *CEMIP* expression (Figures 2B,C, *p* < 0.01). Then, the Breast Cancer Gene-Expression Miner v4.7 resource was employed to confirm the prognostic value of *CEMIP*, with which we further performed survival analysis in ER/PR-positive BC patients and ER/PR-negative BC patients. Based on the DNA microarray data gathered in the Breast Cancer Gene-Expression Miner v4.7 resource, OS, DFS, and DMFS were negatively associated with *CEMIP* expression level (Figures 2D–F, *p* < 0.05) by enrolling all BC patients and OS was also negatively correlated with *CEMIP* expression level in ER/PR-positive BC patients (Figure 2G, *p* < 0.05), whereas no significant correlation existed between patients’ DFS, DMFS, and the expression level of *CEMIP* (Supplementary Figures S3A,B, *p* > 0.05) as well as the OS, DFS, and DMFS in all ER/PR-negative BC patients (Supplementary Figures S3C–E, *p* > 0.05). Finally, we further predicted the prognostic value of *CEMIP* in BC patients by taking the stage, age, molecular subtype, ER/PR/HER2 status, TNM status, and *TP53* mutation status into consideration in addition to the expression level of *CEMIP* using the Cox Proportional Hazards model based on TCGA cohorts. As a result, a total of 789 BC patients were enrolled, and the clinical characteristics and *TP53* state of them are listed in Supplementary Table S1. According to the univariate Cox regression analysis, we found that stage (HR = 2.2, *p* = 2.3e-07), age (HR = 1, *p* = 3.3e-06), node state (HR = 1.5, *p* = 0.00015), tumor states (HR = 1.5, p = 8e-04), metastasis status (HR = 4, *p* = 0.00045), and the expression level of *CEMIP* (HR = 1.1, *p* = 0.042) were significantly negative correlated to OS of BC patients as risk factors (Supplementary Table S2). Next, we included all variables into multivariate analysis and further revealed that only age (HR = 1.05, *p* = 4.18e-07) and the expression level of *CEMIP* (HR = 1.17, *p* = 0.028) were still significantly negatively associated with OS of BC patients in the presence of various factors (Supplementary Table S2).

### DNA Methylation and Genetic Alteration of *CEMIP*

**FIGURE 3:**
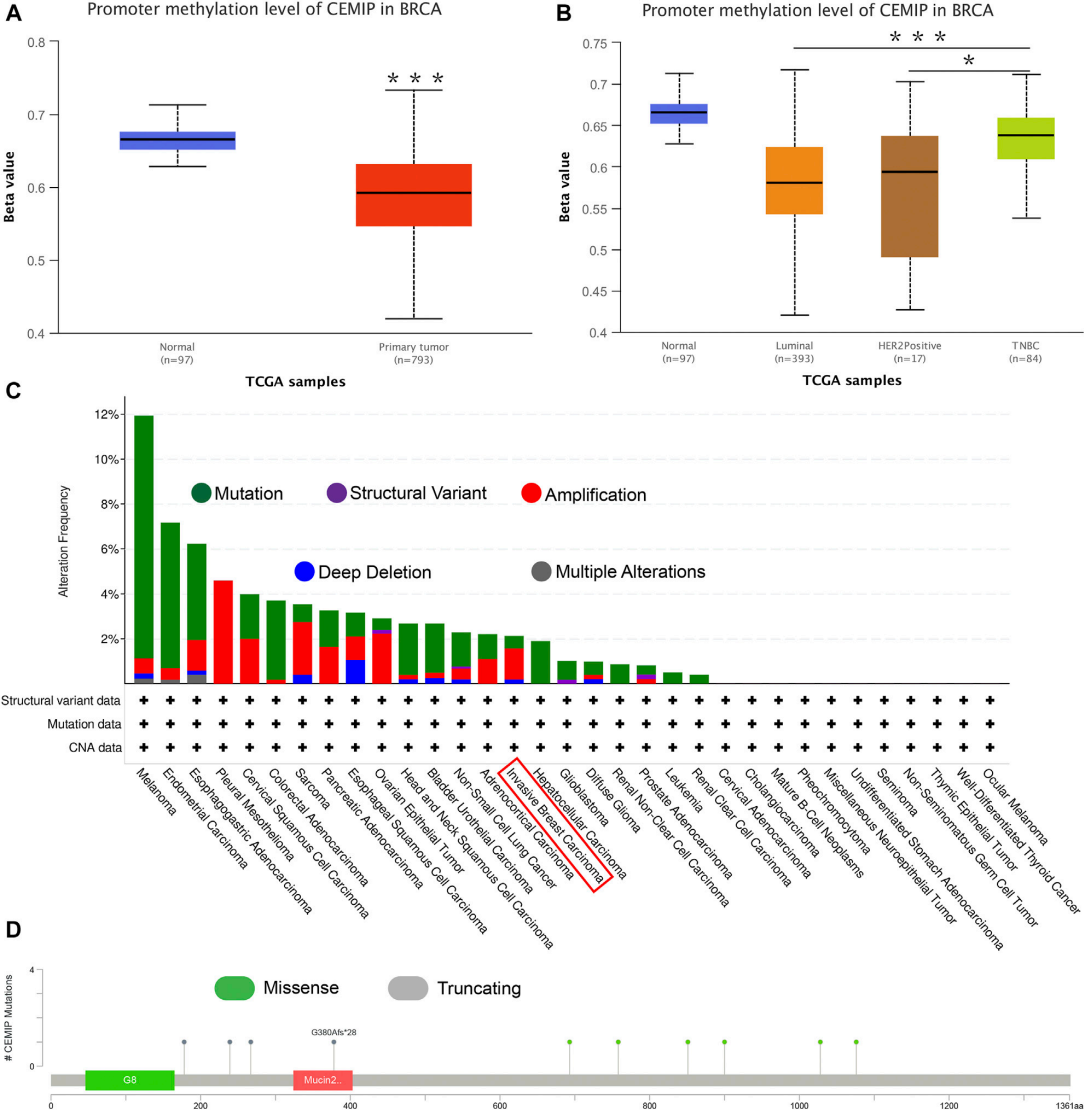
Promoter methylation analysis using the UALCAN web and genetic alteration analysis performed by the cBioPortal web based on TCGA. **(A)** Promoter methylation level of *CEMIP* in BC compared with normal breast samples. ***p < 0.001. **(B)** Promoter methylation level of *CEMIP* in BC subtypes. *p < 0.05; ***p < 0.001. **(C)** Genetic alteration frequency of *CEMIP* in multiple malignancies. **(D)** Mutation site of *CEMIP* in BC.

Next, the UALCAN web was applied to investigate the methylation level of promoter extension of *CEMIP*. As shown in Figure 3, the promoter methylation level of *CEMIP* is significantly lower in BC than in normal tissues (Figure 3A, *p* < 0.001). For subtypes of BC, the promoter methylation level of *CEMIP* was higher in TNBC than in luminal and HER2^+^ BC (Figure 3B, *p* < 0.001 and *p* < 0.05, respectively). Additionally, the cBioPortal resource was employed to assess the genetic alteration of *CEMIP* based on TCGA data. We found that the main genetic alterations of *CEMIP* in various cancers were mutation, amplification, deep deletion, structural variant, and multiple alterations. As for invasive breast carcinoma, the mutation, amplification with the highest alteration frequency, and deep deletion were involved (Figure 3C). The types and sites of *CEMIP* mutation in BC were further explored. As presented in Figure 3D, the missense and truncating are the dominating types and the latter alteration occurs to G380Afs^*^28 of the mucin2 domain with the highest frequency (Figure 3D).

### Predicted Upstream Transcription Factor Targets and Kinase Interactions of *CEMIP*

**FIGURE 4:**
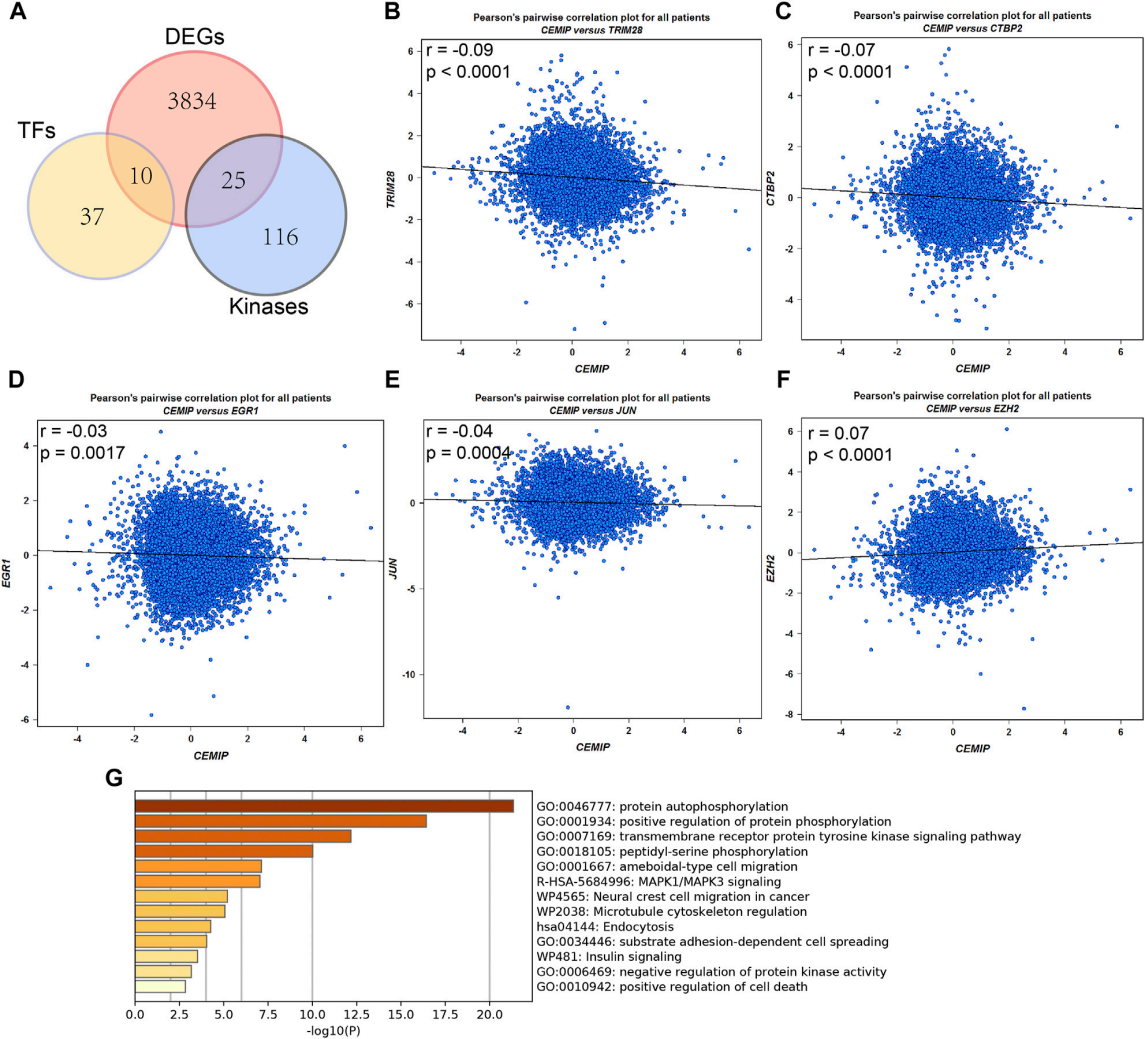
Upstream transcription factor and kinase interaction analysis. **(A)** Differentially expressed predicted upstream TFs of *CEMIP* and kinases interacted with *CEMIP* identified in BC based on DEGs detected from the GSE42568 dataset. **(B–F)** Expression correlations of *CEMIP* and validated TFs in BC. **(G)** Functions and pathways enriched by kinases interacted with *CEMIP* and differentially expressed in BC. DEGs, differentially expressed genes.

**FIGURE 5:**
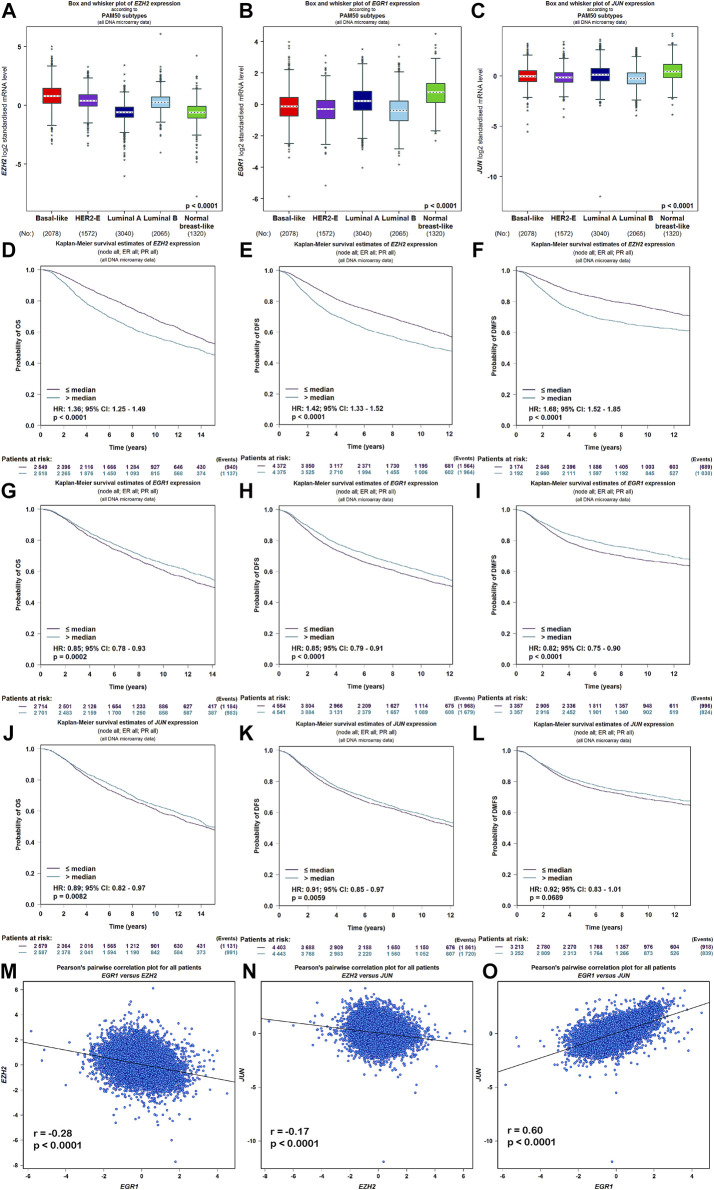
Expression, survival, and correlation analysis of upstream TFs of *CEMIP* in BC. **(A–C)** Expression levels of *EZH2*, *EGR1*, and *JUN* among subtypes of BC. **(D–F)** OS, DFS, and DMFS analysis of *EZH2* in BC patients. **(G–I)** OS, DFS, and DMFS analysis of *EGR1* in BC patients. **(J–L)** OS, DFS, and DMFS analysis of *JUN* in BC patients. **(M–O)** Correlations between *EZH2* and *EGR1*, *EZH2* and *JUN*, and *EGR1* and *JUN* in BC.

Meanwhile, the ARCHS^4^ resource was used to predict upstream TF targets and kinase interactions of *CEMIP.* As a result, a total of 47 unique predicted upstream TFs in humans and 141 kinases were obtained (Figure 4A). Then, we found that 43 TFs existed with genetic alterations in BC with the cBioPortal resource based on TCGA data, among which *ARNT*, *ATF3*, *ESR1*, *TFAP2C*, *TP53*, and *ZNF217* were observed to appear with higher rates of genetic alteration (>5%), and the main alteration type of them was amplification except for *TP53* (Supplementary Figure S4). Furthermore, combined with DEGs identified from the GSE42568 dataset, the expression levels of 10 TFs and 25 kinases were significantly different in BC compared with normal tissues (Figure 4A). Subsequently, we performed validation of their expression through the UALCAN web based on TCGA data as well, and 8 TFs and 22 kinases showed the same results (Supplementary Table S3). For validated TFs, we further evaluated the expression correlations between them and *CEMIP* using the Breast Cancer Gene-Expression Miner based on 11,359 DNA microarrays and found that the expression levels of two up-regulated genes, *TRIM28* and *CTBP2*, and two down-regulated genes, *EGR1* and *JUN*, in BC were negatively associated with *CEMIP*, and one up-regulated gene *EZH2* was positively correlated with *CEMIP* (Figures 4B–F; Supplementary Table S3). Taking expression analysis and correlation analysis into account, we speculated that *EZH2*, *EGR1*, and *JUN* might be the most potential upstream TFs of *CEMIP*. Thereby, expression and survival analyses were further carried out using the Breast Cancer Gene-Expression Miner. Accordingly, *EZH2* was expressed higher in basal-like/TNBC, HER2^+^ and luminal B BC compared to the luminal A BC and normal breast tissues (Figure 5A) and showed negative associations with OS, DFS, and DMFS of BC patients (Figures 5D–F), while the expressions of *EGR1* and *JUN* were lower in these three types of BC (Figures 5B,C) and displayed positive associations with OS, DFS, and DMFS of BC patients (Figures 5G–K) except that the *JUN* expression had no statistically significant correlation with DMFS (Figure 5L). Moreover, the JASPAR^2022^ database was used to predict binding sites of *EGR1*, *JUN*, and *EZH2* in the *CEMIP* promoter region. As shown in Supplementary Table S4, *EGR1* has predicted six binding sites and *JUN* has predicted five binding sites in the *CEMIP* promoter region, whereas *EZH2* could not be retrieved. Subsequently, through literature reviews about *EGR1*, *JUN*, and *EZH2*, we recognized that *EGR1* and *JUN* could be tumor suppressors, whereas *EZH2* can inhibit the expression of tumor suppressors (Baron et al., 2006; Shaulian, 2010; Eich et al., 2020). Therefore, we further assess the correlations among these three TFs. As a result, *EZH2* expression level was negatively correlated with *EGR1* (Figure 5M, r = −0.28, *p* < 0.0001) and *JUN* (Figure 5N, r = −0.17, *p* < 0.0001), while the expression of *EGR1* was positively correlated with *JUN* (Figure 5O, r = 0.60, *p* < 0.0001).

For validated kinases, we carried out enrichment analysis among them and *CEMIP* with the Metascape resource and found that they were mainly enriched in protein autophosphorylation, positive regulation of protein phosphorylation, and the transmembrane receptor protein kinase signaling pathway of GO terms (Figure 4G). Moreover, we integrated all biological processes that *CEMIP* participated in and noticed that two down-regulated genes *DDR2* and *TGFBR2* in BC compared with normal breast tissues and four up-regulated genes, *PTK2*, *RET*, *PRKD2*, and *CEMIP*, were involved in the positive regulation of cell migration, positive regulation of cell motility, positive regulation of cellular component movement, and positive regulation of locomotion (Supplementary Tables S5, S6).

### *CEMIP*-Related Genes and Annotation

**FIGURE 6:**
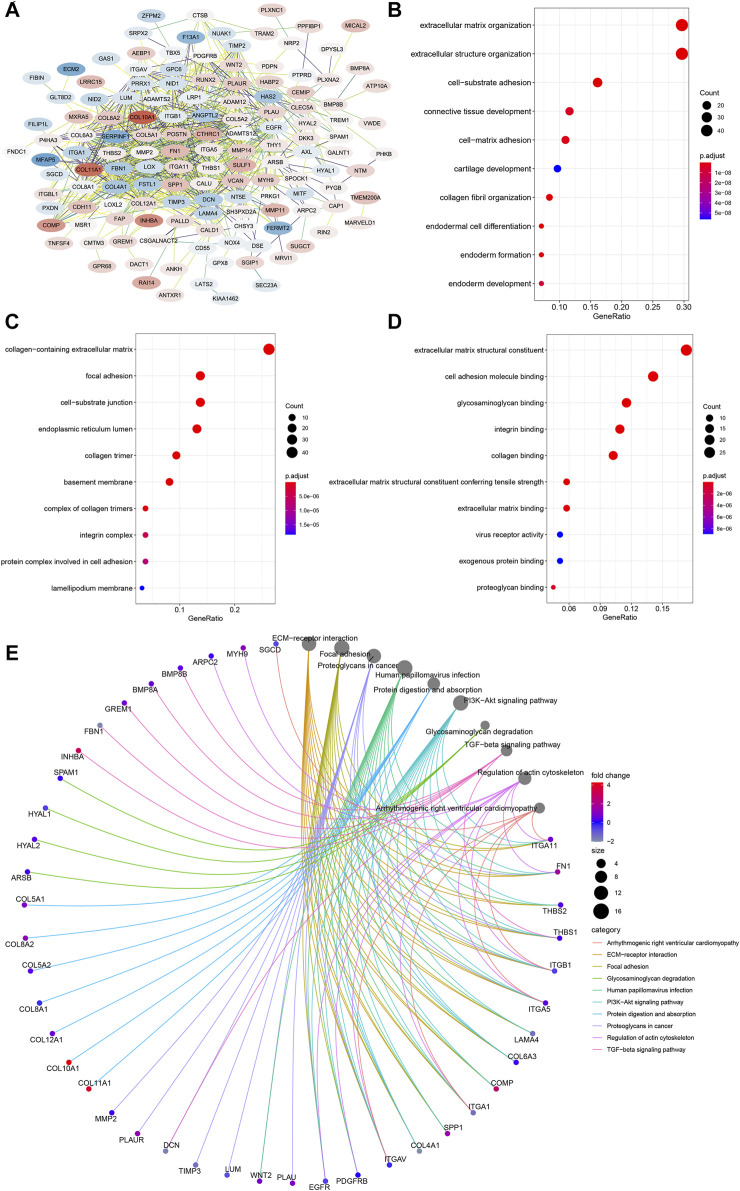
PPI network construction and enrichment analysis of *CEMIP*-related genes. **(A)** PPI network of *CEMIP*-related genes generated by the STRING tool. The red nodes represent up-regulated genes in BC compared with normal samples, while the blue nodes represent down-regulated genes. **(B)** Top 10 BP terms of GO enriched by *CEMIP*-related genes. **(C)** Top 10 CC terms of GO enriched by *CEMIP*-related genes. **(D)** Top 10 MF terms of GO enriched by *CEMIP*-related genes. **(E)** Top 10 KEGG pathways enriched by *CEMIP*-related genes. PPI, protein–protein interaction; GO, Gene Ontology; KEGG, Kyoto Encyclopedia of Genes and Genomes; BP, biological processes; CC, cellular components; MF, molecular functions.

To further inquire about the molecular mechanism of *CEMIP* in BC, we integrated genes that interacted with *CEMIP* obtained from the STRING tool and the genes whose expressions were positively correlated with *CEMIP* expression. Figure 6A shows interacting genes, from which we could observe that the expression levels of most of these genes were distinct in BC compared with normal samples based on the DEG analysis of the GSE42568 dataset. Furthermore, gene annotation of GO functions, including biological process (BP), cellular component (CC), and molecular function (MF), and the KEGG pathway was executed by R package clusterProfiler. The top 10 terms of them are exhibited in Figures 6B–E. Most of these genes participated in the extracellular matrix organization, extracellular structure organization, and cell-substrate adhesion of BP (Figure 6B); located in the collage-containing extracellular matrix, focal adhesion, cell-substrate junction, and endoplasmic reticulum lumen of CC (Figure 6C); possessed extracellular matrix structural constitutes, cell adhesion molecule binding, glycosaminoglycan binding, integrin binding and collagen binding of MF (Figure 6D); and were involved in ECM–receptor interaction, focal adhesion, the PI3K-Akt signaling pathway, and the TGF-beta signaling pathway (Figure 6E).

### Correlations of *CEMIP* Expression With the Immune Cell Infiltration Level in Cancers

**FIGURE 7:**
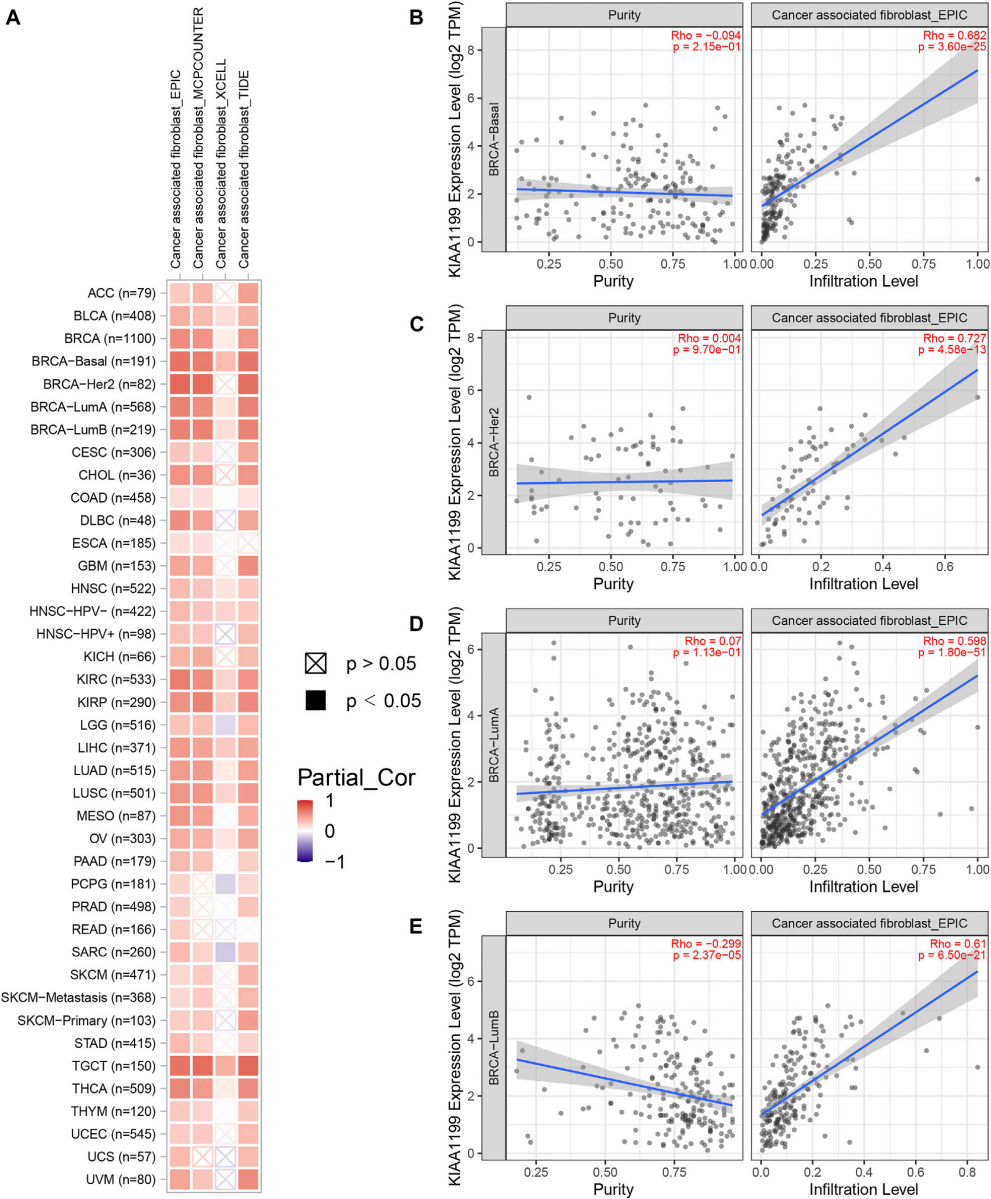
Correlation of *CEMIP* expression with CAF infiltration level explored by TIMER2.0 web. **(A)** Correlations between *CEMIP* expression and the infiltration level of CAF across various types of cancers analyzed by four algorithms provided (EPIC, MCPCOUNTER, XCELL, and TIDE). The red squares represent positive correlations, while the blue squares represent negative correlations with statistical significance (*p* < 0.05). **(B–E)** Correlations between *CEMIP* expression and the infiltration level of CAF among four subtypes of BC (basal-like/TNBC, HER2^+^, luminal A, and luminal B) using the EPIC algorithm. CAF, cancer-associated fibroblast.

Given that the tumor immune microenvironment (TIM) plays an important role in the progress and metastasis of cancers, we evaluated the correlations of *CEMIP* expression with several immune cell infiltration levels in various types of cancers, consisting of CD8^+^ T cells, CD4^+^ T cells, B cells, macrophages, neutrophils, DC, NK cells, and CAF using the TIMER2.0 web with all algorithms provided. Generally, the infiltration of macrophages, neutrophils, resting NK cells, and CAF was positively related to *CEMIP* expression with all or most algorithms in most carcinomas, including BC (Supplementary Figures S5, S6A and Figure 7A), while activated NK cells, CD8^+^ T cells, and CD4^+^ Th1 cells were opposite (Supplementary Figures S6, S7A) and other immune cell (that is., CD4^+^ T cells, B cells, and DC) infiltrations showed no clear unifying trends with these available algorithms (Supplementary Figures S7, S8). We noticed that the infiltration of B cells was negatively associated with the expression level of *CEMIP* in BC (Supplementary Figure S7B). We also noted that the CAF infiltration had the highest correlations with *CEMIP* expression in BC, the scatterplots of which among subtypes of BC generated using the EPIC algorithm are presented in Figures 7B–E.

### Expression Correlations of *CEMIP* With Biomarkers of Immune Cells and Checkpoints in BC

To further estimate the role of *CEMIP* in BC immune regulation, we explored the expression correlations of *CEMIP* with biomarkers of immune cells whose infiltration levels were significantly associated with *CEMIP* expression and immune checkpoints in BC. As listed in Table 1, *CEMIP* had significant positive correlations with M1 macrophages’ biomarkers *NOS2* (r = 0.07, *p* < 0.0001) and *IRF5* (r = 0.12, *p* < 0.0001); M2 macrophages’ biomarkers *CD163* (r = 0.23, *p* < 0.0001), *VSIG4* (r = 0.24, *p* < 0.0001), and *MS4A4A* (r = 0.17, *p* < 0.0001); neutrophils’ biomarker *ITGAM* (r = 0.22, *p* < 0.0001); and immune checkpoints PD-L1 (r = 0.08, *p* < 0.0001) and CTLA4 (r = 0.06, *p* < 0.0001) and negative correlations with CD8^+^ T cells’ biomarker *CD8A* (r = −0.02, *p* = 0.0331) and neutrophils’ biomarker *CCR7* (r = −0.06, *p* < 0.0001).

**TABLE 1:** Expression correlations of *CEMIP* with biomarkers of immune cells and immune checkpoints.

Immune cell	Biomarker	r value	*p*-value
B cell	CD19	−0.02	0.0558
	CD79A	−0.00	0.8227
CD8^+^ T cell	CD8A	−0.02^a^	0.0331*
	CD8B	−0.02	0.0611
M1 macrophage	NOS2	0.07^a^	<0.0001****
	IRF5	0.12^a^	<0.0001****
	PTGS2	−0.01	0.5330
M2 macrophage	CD163	0.23^a^	<0.0001****
	VSIG4	0.24^a^	<0.0001****
	MS4A4A	0.17^a^	<0.0001****
Neutrophil	CEACAM8	0.00	0.6818
	ITGAM	0.22^a^	<0.0001****
	CCR7	−0.06^a^	<0.0001****
Checkpoints	PDCD1 (PD1)	−0.01	0.3018
	CD274(PD-L1)	0.08^a^	<0.0001****
	CTLA4	0.06^a^	<0.0001****

a These results are statistically significant. *p-value < 0.05; **p-value < 0.01; ***p-value < 0.001; ****p-value < 0.0001.

### KEGG Pathway and HALLMARK Aberrations Correlated With CEMIP in BC

**FIGURE 8:**
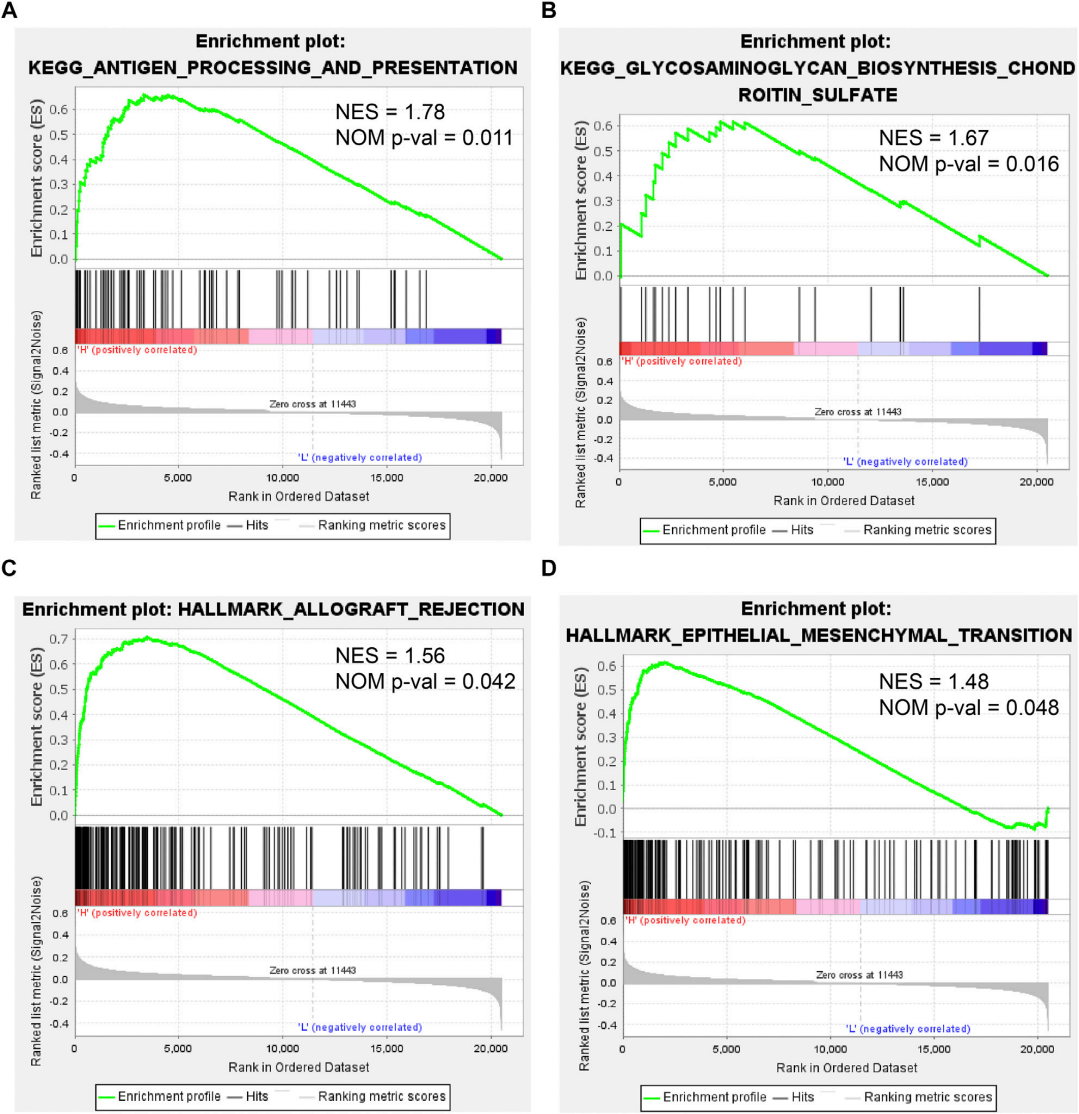
Gene Set Enrichment Analysis based on the GSE42568 dataset and the gene sets of KEGG pathways and HALLMARK. **(A,B)** Top two KEGG pathways enriched by the *CEMIP* high expression group with the highest NES and NOM *p*-val < 0.05. **(C,D)** Two HALLMARK terms enriched by the *CEMIP* high-expression group with NOM *p*-value < 0.05. NES, normalized enrichment score; NOM *p*-value, nominal *p*-value.

To further investigate the effect of *CEMIP* on BC, we manipulated Gene Set Enrichment Analysis according to the expression of *CEMIP* based on the gene expression matrix of the GSE42568 dataset and gene sets of the KEGG pathway and HALLMARK. As listed in Supplementary Table S7, a total of 10 gene sets were up-regulated in the high-expression group of *CEMIP* and were significantly enriched at nominal *p*-value < 0.05 based on the KEGG pathway, while two gene sets were based on HALLMARK. The top two KEGG pathways and HALLMARK processes enriched were “ANTIGEN_PROCESSING_AND_PRESENTATION, GLYCOSAMINOGLYCAN_ BIOSYNTHESIS_CHONDROITIN_SULFATE” of KEGG” (Figures 8A,B) and “ALLOGRAFT_REJECTION, EPITHELIAL_MESENCHYMAL_TRANSITION” (Figures 8C,D), respectively.

## Discussion

To date, BC has been the most frequently diagnosed malignancy worldwide and leads to 684,996 deaths (Rodrigues et al., 2019; Zhai et al., 2020). Although the prognosis of cancer patients has improved dramatically with the advanced medical technology (Rodrigues et al., 2019; Zhai et al., 2020), a complete cure for BC patients is not yet possible with the currently available therapies due to the ambiguous mechanism of tumorigenesis and tumor progression in BC. Accumulated evidence shows that the over-expression of *CEMIP* could enhance proliferation, adhesion, motility, invasiveness, and EMT of various carcinomas, including BC, as well as its transcriptional regulation mechanism in many cancers (Liu et al., 2021a), indicating that *CEMIP* might play an important role in BC. Therefore, we performed an integrated analysis of *CEMIP* in BC and other cancers using bioinformatics approaches and a variety of online analysis tools mainly based on TCGA and GEO databases.

CEMIP is a type of secreted protein in exosomes and also exists in normal human tissues, like the brain, lung, pancreas, and testis (Zhai et al., 2020; Rodrigues et al., 2019). It has been described that CEMIP could mediate depolymerization of hyaluronic acid (HA) (Yoshida et al., 2013), bind to HA, hydrolyze high-molecular-weight HA, and be involved in hyaluronan catabolism as summarized in https://www.uniprot.org/uniprot/Q8WUJ3. In the present study, we also explored CEMIP expression in normal tissues, immune cells, and its subcellular locations. According to previous findings, CEMIP can be detected in normal tissues, including plasma, and even in immune cells, which mainly are located in the plasma membrane, extracellular regions, nucleus, and endoplasmic reticulum. It was reported that over-expression of CEMIP in exosomes might facilitate the metastasis of BC (Rodrigues et al., 2019; Zhai et al., 2020). Other research studies revealed that CEMIP residing in the endoplasmic reticulum may enhance BC cell survival in hypoxia and cancer cell migration by upregulating and interacting with binding immunoglobulin protein (Evensen et al., 2013; Banach et al., 2019). Thereby, the over-expression of CEMIP detected in blood might be a convenient way to early diagnose BC metastasis. However, targeting CEMIP therapy for BC patients might be more complicated in the consideration of its normal biological functions, which acquires a precise delivery route, like targeting CEMIP located in the endoplasmic reticulum.

Subsequently, we conducted a pan-cancer analysis of *CEMIP* expression based on the TCGA database and discovered that it was highly expressed in most cancers, consistent with early studies (Evensen et al., 2013; Jami et al., 2014; Zhang et al., 2014; Fink et al., 2015; Shen et al., 2019; Li et al., 2020a; Zhai et al., 2020; Liu et al., 2021a; Chen et al., 2021), after which we focused on *CEMIP* expression in BC. However, we did not observe a statistically significant difference in *CEMIP* expression based on BC subtypes (luminal, HER2^+^, and TNBC), individual cancer stages, patient age, and nodal metastasis status except for the *TP53* mutation status at first. Then, the Breast Cancer Gene-Expression Miner v4.7 resource, which integrated almost all public BC data comprising DNA microarrays and RNA-seq, was employed to evaluate *CEMIP* expression based on BC subtypes, patient age, nodal metastasis status, and *TP53* mutation status again with 11,359 DNA microarrays. We noticed that *CEMIP* was highly expressed in HER2^+^ and TNBC compared with luminal type and was expressed higher in the group of patients with an age over 51 years than those with an age less than 51, suggesting that *CEMIP* could be an indicator of aggressive types of BC, like HER2^+^ and TNBC, and might be responsible for some characteristics of invasive BC. In addition, the inconsistency of results from the two web tools in our findings might be primarily caused by the sample size. Several studies described that *CEMIP* was highly expressed in invasive breast cancer specimens and in invasive MDA-MB-231 TNBC cell lines, whereas some researchers observed much lower expression of *CEMIP* in non-invasive BC cells with a low-invasive potential, like MCF-7, T-47D, and ZR-75-1 cell lines (Evensen et al., 2013; Zhang et al., 2014). Of note, *CEMIP* was expressed lower in samples without *TP53* mutation than *TP53* mutation samples with one accord using these two webs and databases. *TP53* is a well-known cancer suppressor, whose mutation has been reported in plenty of malignancies (Miller et al., 2005). Our finding shows that the high expression of *CEMIP* in invasive BC might be partly caused by *TP53* mutation since it has been reported that TNBC exhibits more *TP53* mutation (Verigos and Magklara, 2015). To our knowledge, no study has yet linked them.

After identifying the discrepancy of *CEMIP* expression in BC, we further investigated its prognostic value among BC patients. We found that the expression of *CEMIP* was negatively associated with OS, RFS, DFS, and DMFS of BC patients when all types of BC were included. However, when we divided BC patients into ER^+^/PR^+^ and ER^−^/PR^−^ groups, only OS of BC patients with ER^+^/PR^+^ was significantly negatively correlated with *CEMIP* expression, indicating that its prognostic prediction in BC patients with ER^+^/PR^+^ might be more significant since the patients with TNBC have been widely proved to have poor prognoses (Waks and Winer, 2019). More convincingly, we discovered that high expression of *CEMIP* had a strong relationship with increased risk of death in BC patients according to both univariate and multivariate Cox regression analysis with hazard ratio (HR) = 1.17 and *p*-value = 0.028 of the latter, that is, holding the other covariates, that is., BC patient stage, age, ER/PR/HER2 status, TNM status, and *TP53* mutation status, constant, a higher expression value of *CEMIP* predicted a poor survival of BC patients. Given these results, *CEMIP* might be a promisingly prognostic and therapeutic target for BC patients.

It has been reported that *CEMIP* mutation in the GG domain leads to non-syndromic hearing loss, while its over-expression prevailingly contributes to its oncogenic roles (Liu et al., 2021a). However, in this study, we found that the genetic alterations of *CEMIP* appeared in multiple cancers, including invasive BC, suggesting that mutations (that is, missense and truncating) of *CEMIP* may be responsible for the formation and progression of aggressive BC, especially truncating in the G380Afs^*^28 of mucin2 domain of *CEMIP*. Although studies found that *CEMIP* mutation happens in invasive BC cell lines, like MDA-MB-435 and MDA-MB-231 (Zhang et al., 2014), the mechanism of this needs more experiments to explore.

Kuscu et al. (2012) elucidated that the regulatory mechanisms which control *CEMIP* expression were genetic and epigenetic. In agreement with the previous study, we discovered a link between DNA hypomethylation and high expression of *CEMIP*. Furthermore, we also identified some predicted upstream TFs mutated or aberrantly expressed in BC. The result again suggested that *CEMIP* expression might be influenced by *TP53* mutation with the highest mutation frequency. Except for *TP53* mutation*, ARNT*, *ATF3*, *ESR1*, *TFAP2C*, and *ZNF217* alterations might also affect the expression of *CEMIP.* Meanwhile, we found three potential up-regulated genes (*TRIM28*, *CTBP2*, and *EZH2*) and two down-regulated genes (*EGR1* and *JUN*) in BC compared with the normal breast tissues which might regulate *CEMIP* expression, especially *EZH2*, *EGR1*, and *JUN*, due to the fact that the correlations between their expression levels and *CEMIP* expression were consistent with the directions of change. That is, the up-regulated gene *EZH2* showed a positive correlation with *CEMIP*, and the down-regulated genes *EGR1* and *JUN* had negative correlations with *CEMIP*, indicating that the abnormal expression levels of these three TFs were likely to be responsible for *CEMIP* over-expression in BC. Moreover, *EGR1* and *JUN* were included in the JASPAR^2022^ database, where they had six and five possible binding sites in the *CEMIP* promoter region, respectively. Additionally, the high expression of *EZH2* predicted low OS, DFS, and DMFS of BC patients, while *EGR1* and *JUN* were opposite. *JUN*, a member of AP-1, is a controversial gene in cancer, which could be an oncogene or a tumor suppressor (Shaulian, 2010). It was reported to regulate *CEMIP* expression but as an activator of the *CEMIP* promoter (Shaulian, 2010). Hence, the specific circumstance needs more experiments to find out. *EGR1* is a cancer suppressor and has been confirmed to be down-regulated in BC compared with normal tissues (Baron et al., 2006), but no studies revealed the regulatory relationship between it and *CEMIP*. *EZH2* is classified as an oncogene, shows high expression in numerous cancers including breast cancer, and is discovered to contribute to global transcriptional repression, mainly targeting tumor suppressor genes (Eich et al., 2020). However, no studies revealed the regulatory relationship between it and *CEMIP* either. Then, given that *EZH2* can inhibit the expression of tumor suppressors, we further assess the correlations among these three TFs. As expected, the *EZH2* expression level was negatively correlated with *EGR1* and *JUN*, while the expression of *EGR1* was positively correlated with *JUN*, suggesting that *EZH2*/*EGR1* and *JUN*/*CEMIP* might be the potential regulatory pathways in BC, especially in invasive BC, like TNBC and HER2^+^ BC, or *EZH2*, *EGR1*, and *JUN* mediate *CEMIP* expression directly in BC.

Cancer invasion and metastasis are basically dependent on cell migration (Hanahan and Weinberg, 2011; Evensen et al., 2013). It has been demonstrated that *CEMIP* can promote prostate, breast, and colon cancer cell motility (Evensen et al., 2013). In our study, we first identified differentially expressed kinases that interacted with *CEMIP* in BC and performed enrichment analysis on these genes, including *CEMIP*. We noted that *DDR2*, *PTK2*, *RET*, *TGFBR2*, *PRKD2*, and *CEMIP* were involved in positive regulation of cell migration, indicating that *CEMIP* might participate in cancer cell migration by interacting with these genes, of which *DDR2* and *TGFBR2* were down-regulated in BC and proved to inhibit cancer metastasis (Lo Sardo et al., 2021; Mehta et al., 2021), while *PTK2*, *RET*, and *PRKD2* were up-regulated in BC and were shown to promote cancer development (Azoitei et al., 2014; Fan et al., 2019; Subbiah and Cote, 2020). However, no studies have revealed the relationships between them yet. Moreover, we also integrated *CEMIP*-related genes and found them mainly enriched in the ECM–receptor interaction, focal adhesion, the PI3K-Akt signaling pathway, and the TGF-beta signaling pathway, suggesting that *CEMIP* might promote cancer cell invasion and metastasis through these pathways. Some studies reported that *CEMIP* is involved in EMT, Wnt/β-catenin, MEK/ERK, and PI3K/Akt signal pathways to promote cancer progression, while the exact mechanism is still ambiguous.

In the present study, we first evaluated the correlations of the expression of *CEMIP* with the infiltration levels of immune cells and found that biomarkers of these infiltrated immune cells were significantly related to *CEMIP* expression as well as three well-known immune checkpoints. Through rigorous evaluation of a variety of algorithms, we observed that the infiltration of macrophages, neutrophils, resting NK cells, and CAF was positively related to *CEMIP* expression in most carcinomas, including BC, while activated NK cells and CD8^+^ T cells were opposite, and the infiltration of B cells was negatively associated with the expression level of *CEMIP* in BC. Moreover, we discovered that *CEMIP* was correlated with some biomarkers of these immune cells and immune checkpoints, especially, *CD163* and *VSIG4* of M2 macrophages, and *ITGAM* of neutrophils. It has been widely accepted that the body’s immune system plays a dual role in tumor initiation and progression, which suppress tumor growth in the early phase of oncogenesis, but promoting tumor progression once a tumor becomes invasive (Schreiber et al., 2011). The TME, consisting of the extracellular matrix (ECM), stromal cells (such as fibroblasts), and immune cells (comprising T and B lymphocytes, NK cells, and tumor-associated macrophages), provides mechanical support for the tumor (Roma-Rodrigues et al., 2019). Additionally, accumulated evidence has documented that the immune cell infiltration in TME is associated with BC patient outcomes. For instance, the high level of lymphocytic infiltration may predict a better prognosis in patients with early-stage TNBC and HER2^+^ BC (Savas et al., 2016; Gao et al., 2020), whereas the infiltration of CAF and M2 macrophages may contribute to cancer progression (Ishii et al., 2016; Xia et al., 2020)*.* Combining these existing research studies and our findings, *CEMIP* might promote the occurrence and development of tumors via participating in the formation of TME including both ECM and the immune microenvironment (immune cells), indicating that *CEMIP* may be a promisingly therapeutic target for advanced BC.

Finally, we performed GSEA according to the expression of *CEMIP* based on KEGG pathways and HALLMARK. In the group with high expression of *CEMIP,* the pathways and hallmarks significantly enriched were related to antigen presentation and EMT, which again revealed the entanglement of *CEMIP* with the EMT pathway and tumor immune infiltration.

In conclusion, we illustrated that *CEMIP* was highly expressed in various kinds of carcinomas, including BC, especially advanced subtypes, and predicted less favorable prognosis (negatively associated with OS, RFS, DFS, and DMFS) in BC patients, and the higher the expression of it, the worse the outcomes BC patients have. We revealed that the mutation and high expression of *CEMIP* might lead it to an oncogene. We also demonstrated that *TP53* mutation, DNA hypomethylation, and the expression changes of upstream TFs of *CEMIP* were likely to cause hyper-expression of *CEMIP*, and we further identified three potential upstream TFs in BC, namely, *EZH2*, *EGR1*, and *JUN.* Moreover, our findings suggested that *CEMIP* was closely related to TME and might exert its oncogenic roles by participating in the extracellular matrix formation, mainly increasing CAF, M2 macrophage, and neutrophil infiltration and decreasing CD8^+^ T cell infiltration. Of course, these findings need more solid confirmations of further experiments and clinical trials in the future.

## Data Availability

The datasets presented in this study can be found in online repositories. The names of the repository/repositories and accession number(s) can be found in the article/Supplementary Material.
