# Supplementary material for: High Expression of CEMIP Correlates Poor Prognosis and the Tumur Microenvironment in Breast Cancer as a Promisingly Prognostic Biomarker
Source: Front Genet. 2021 Dec 13;12:768140. doi: 10.3389/fgene.2021.768140 (PMC8710689; doi:10.3389/fgene.2021.768140)
Supplement: Supplementary file 1 [file DataSheet1.PDF]

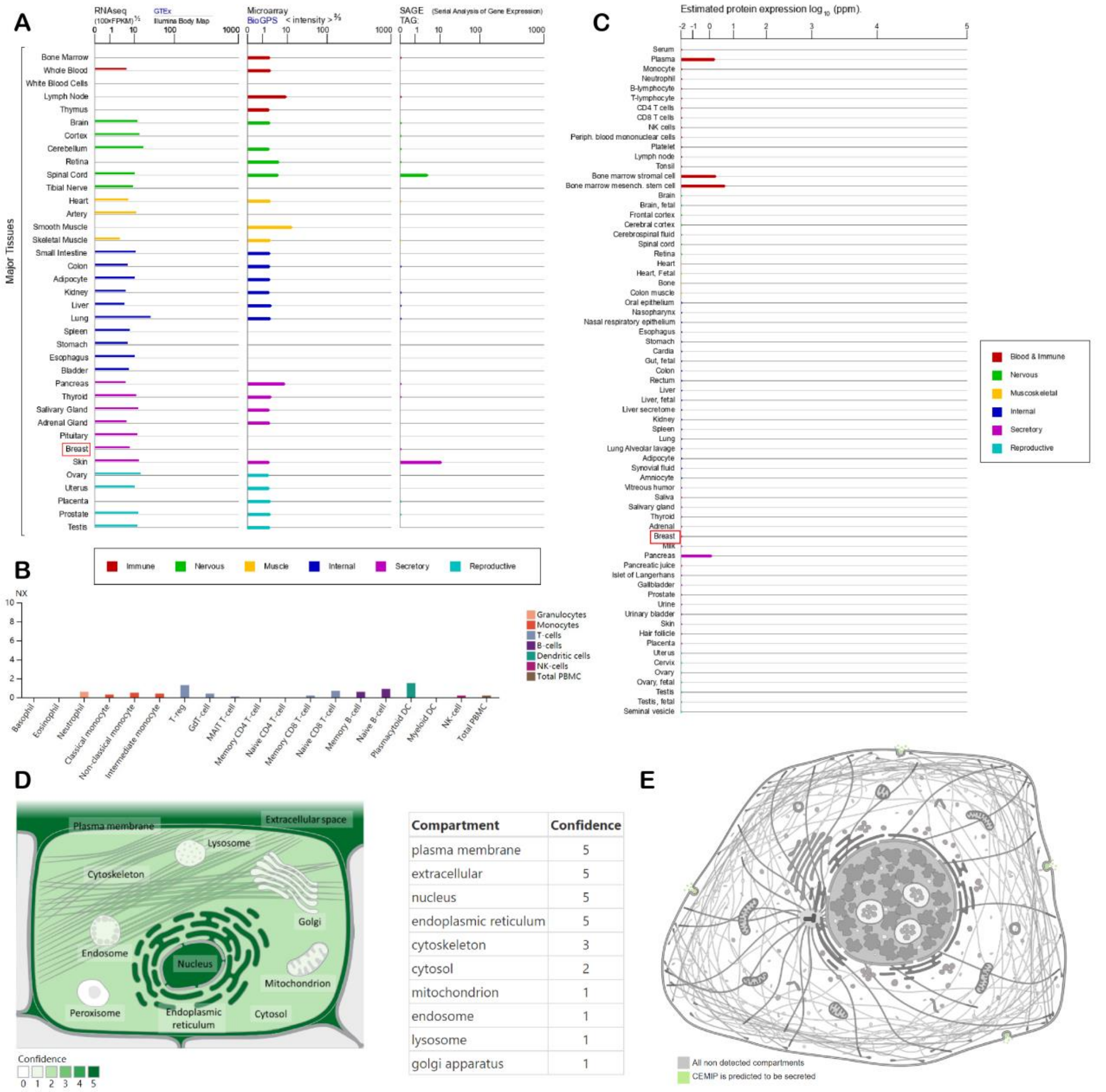

Compartment	Confidence
plasma membrane	5
extracellular	5
nucleus	5
endoplasmic reticulum	5
cytoskeleton	3
cytosol	2
mitochondrion	1
endosome	1
lysosome	1
golgi apparatus	1

FIGURE S1 CEMIP expression in normal tissues, immune cells and cellular localization obtained from GeneCards®: The Human Gene Database and The Human Protein Atlas. **(A)** The mRNA expression level of *CEMIP* in major normal tissues. **(B)** *CEMIP* expression in a variety of immune cells. **(C)** The estimated protein expression of CEMIP in multiple normal tissues. **(D-E)** CEMIP subcellular localization.

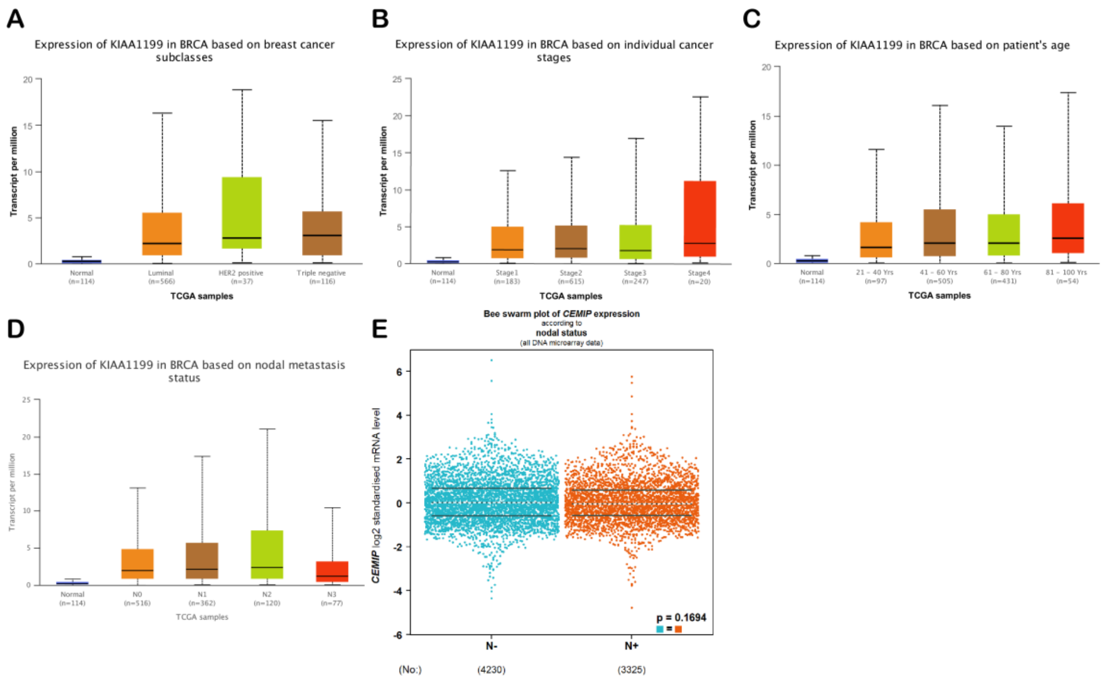

FIGURE S2 Expression analysis of *CEMIP* based on clinical features of BC. **(A-D)** The expression of *CEMIP* based on subtype of BC, individual cancer stage, patient's age and nodal metastasis status analyzed by UALCAN web using TCGA database without statistical significance ($p > 0.05$). **(E)** The expression of *CEMIP* based on nodal metastasis status analyzed by the Breast Cancer Gene-Expression Miner v4.7 resource with 11359 DNA microarrays. TCGA, The Cancer Genome Atlas; *KIAA1199, CEMIP*.

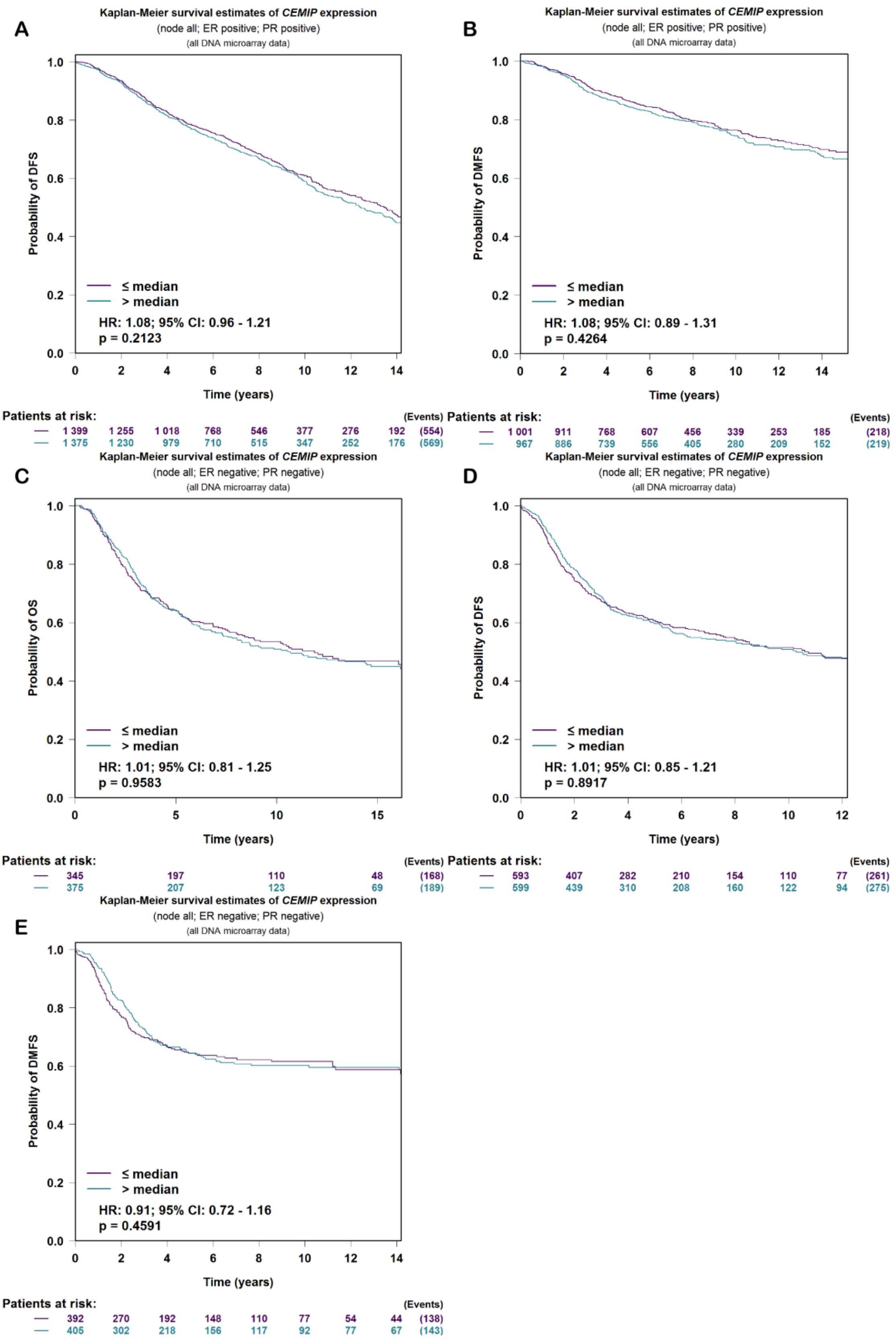

FIGURE S3 Survival analysis performed by the bc-GenExMiner tool. **(A-B)** The DFS and DMFS plots of *CEMIP* in ER/PR+ BC. **(C-E)** The OS, DFS and DMFS plots of *CEMIP* in ER/PR- BC subtypes. The bc-GenExMiner, the Breast Cancer Gene-Expression Miner v4.7.

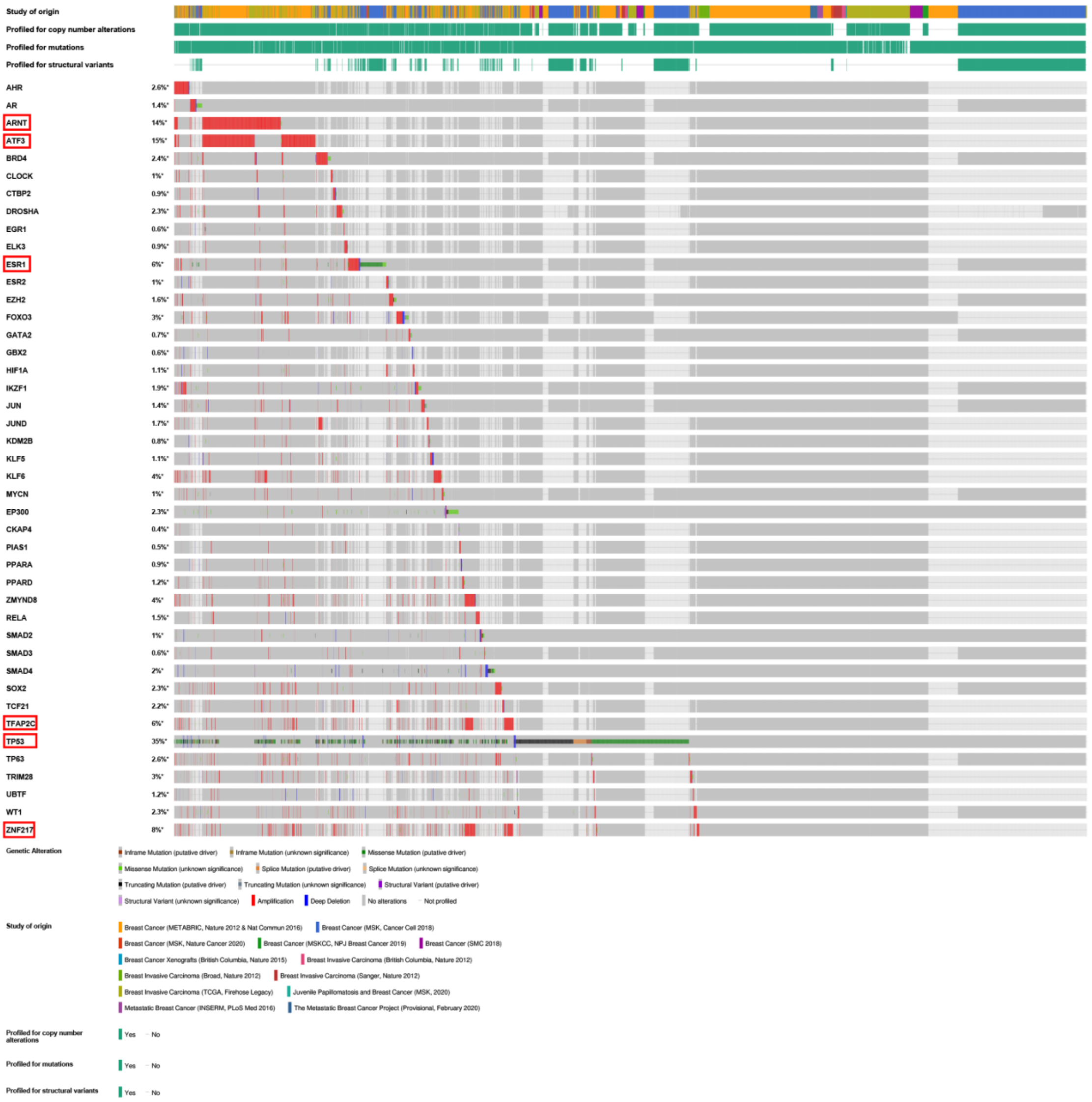

FIGURE S4 Genetic alteration analysis of predicted upstream TFs performed by the cBioPortal web based on TCGA. The genes framed in red with alteration frequency > 5%. TFs, transcription factors.

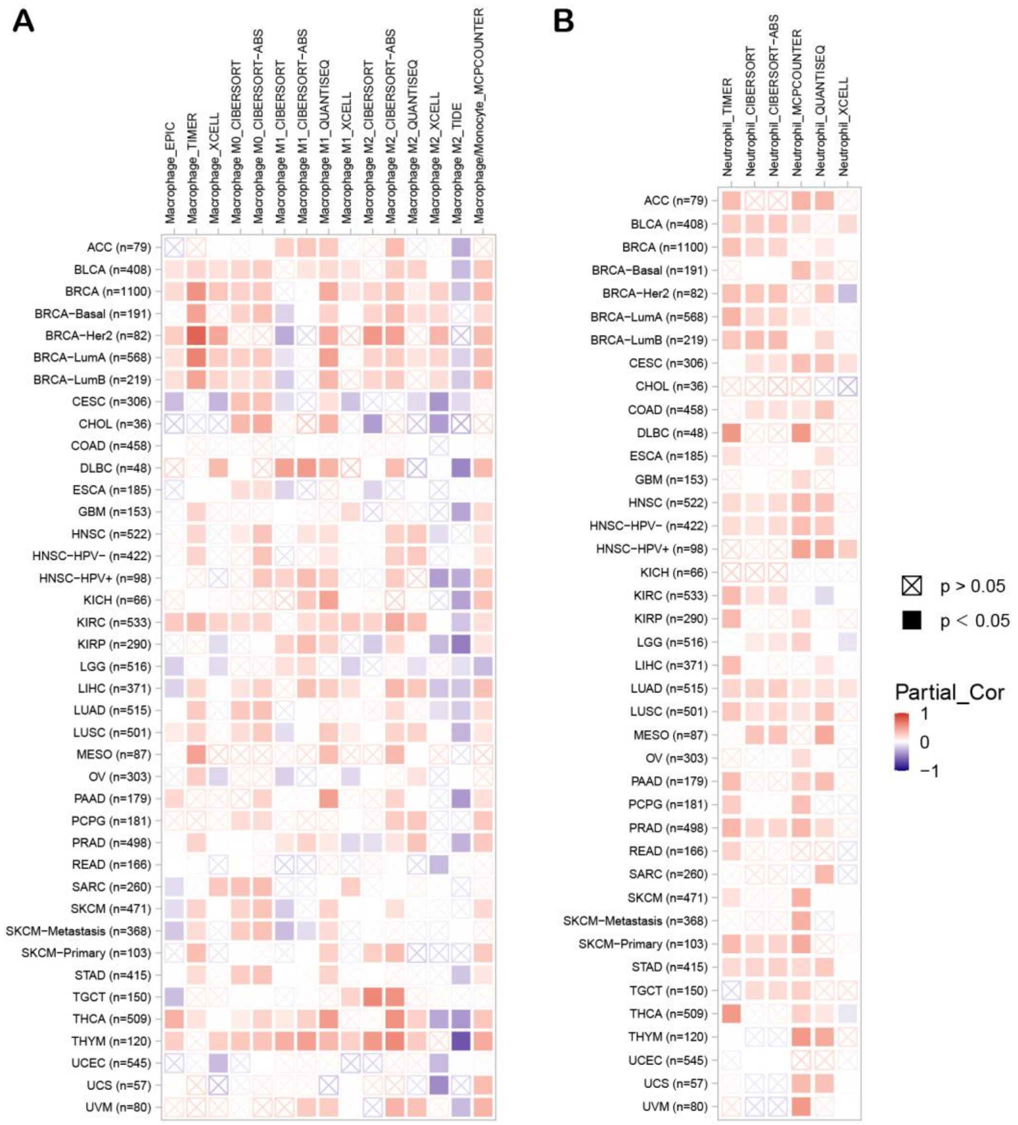

FIGURE S5 The Correlations of *CEMIP* expression with macrophage **(A)** and neutrophil **(B)** infiltration level across various types of cancers analyzed by all algorithms provided in TIMER2.0 web. The red squares represent positive correlations, while the blue squares represent negative correlations with statistical significance ($p < 0.05$).

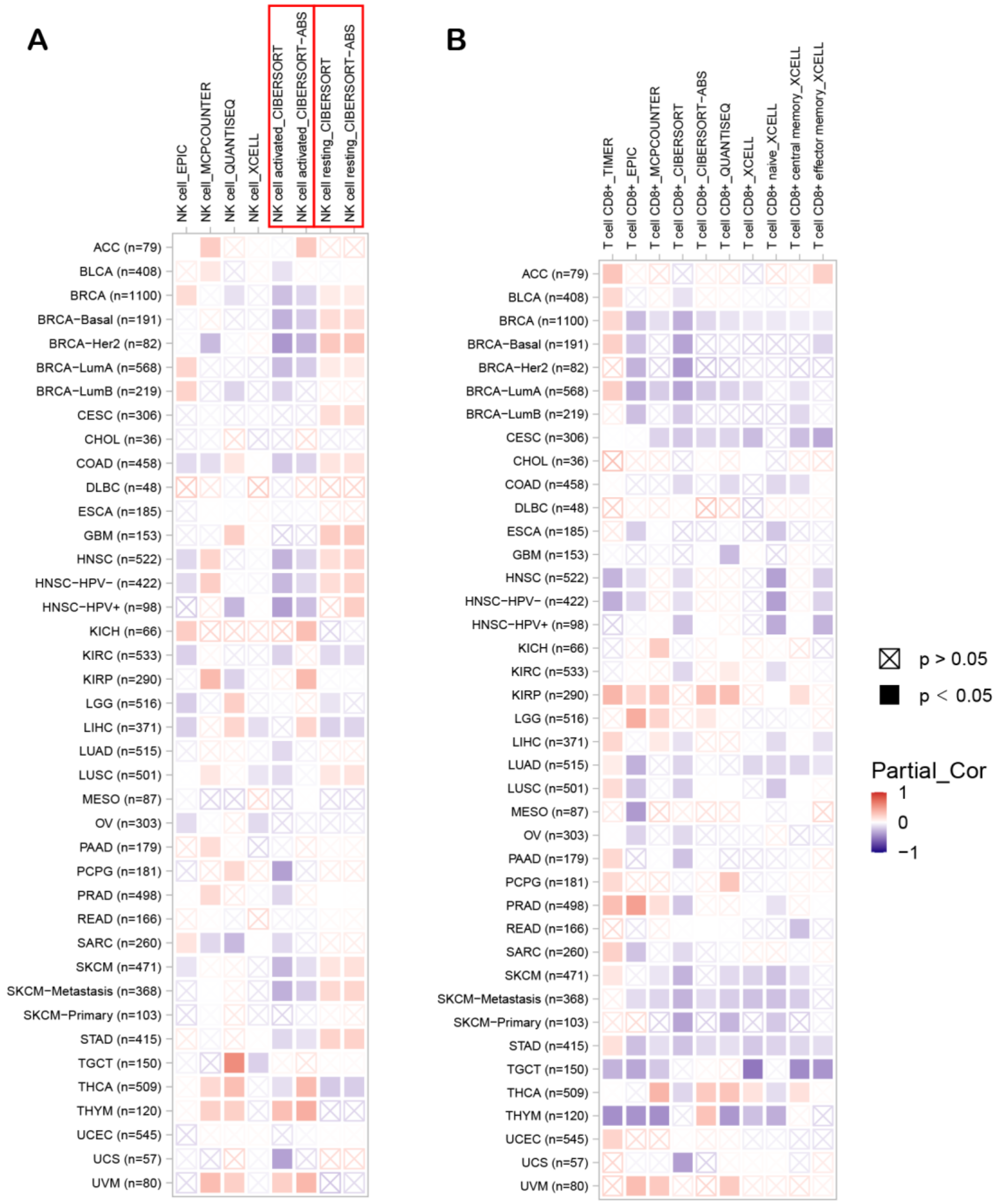

FIGURE S6 The Correlations of *CEMIP* expression with NK cell **(A)** and CD8+ T cell **(B)** infiltration level across various types of cancers analyzed by all algorithms provided in TIMER2.0 web. The red squares represent positive correlations, while the blue squares represent negative correlations with statistical significance ($p < 0.05$). NK, natural killer.

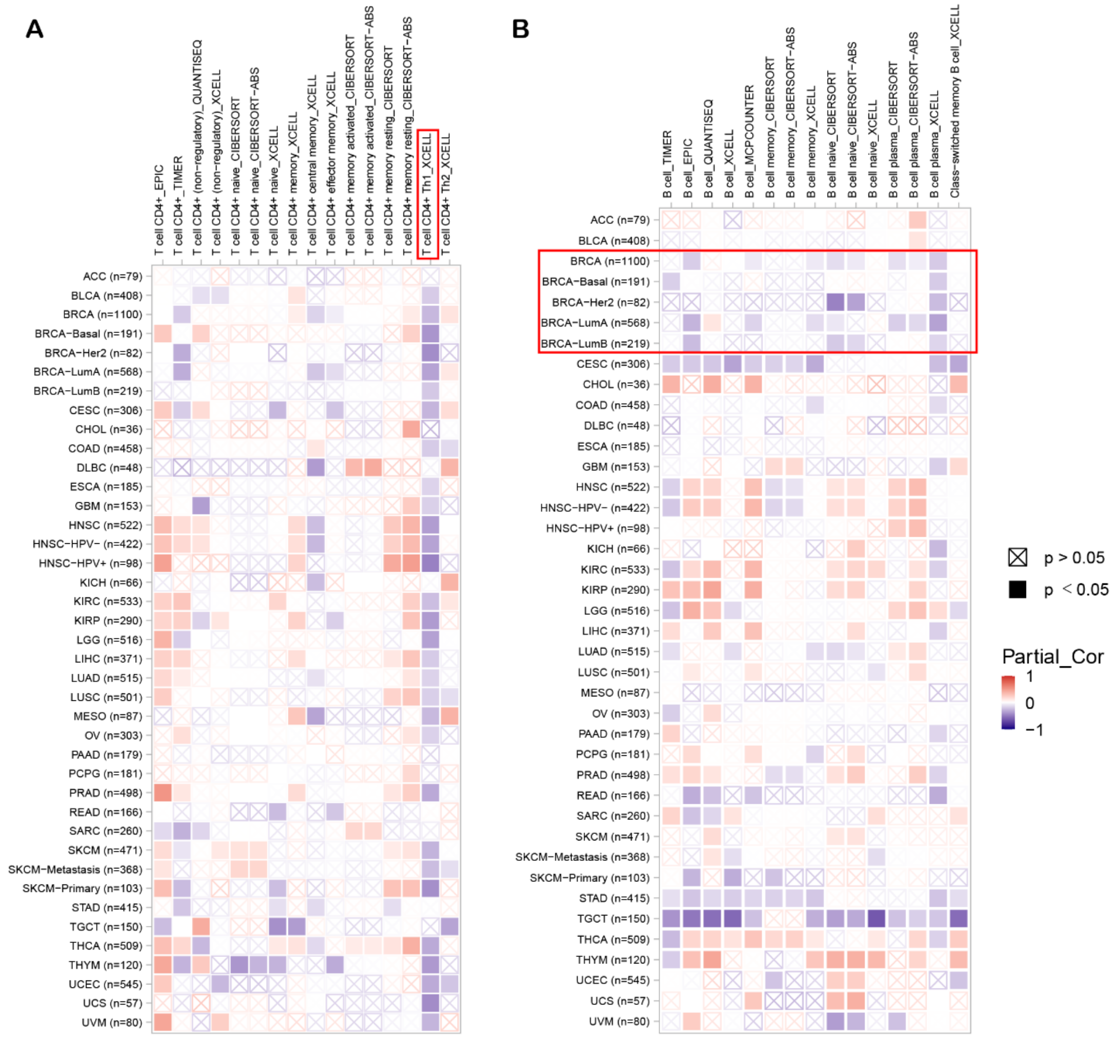

FIGURE S7 The Correlations of *CEMIP* expression with CD4+ T cell **(A)** and B cell **(B)** infiltration level across various types of cancers analyzed by all algorithms provided in TIMER2.0 web. The red squares represent positive correlations, while the blue squares represent negative correlations with statistical significance ($p < 0.05$).

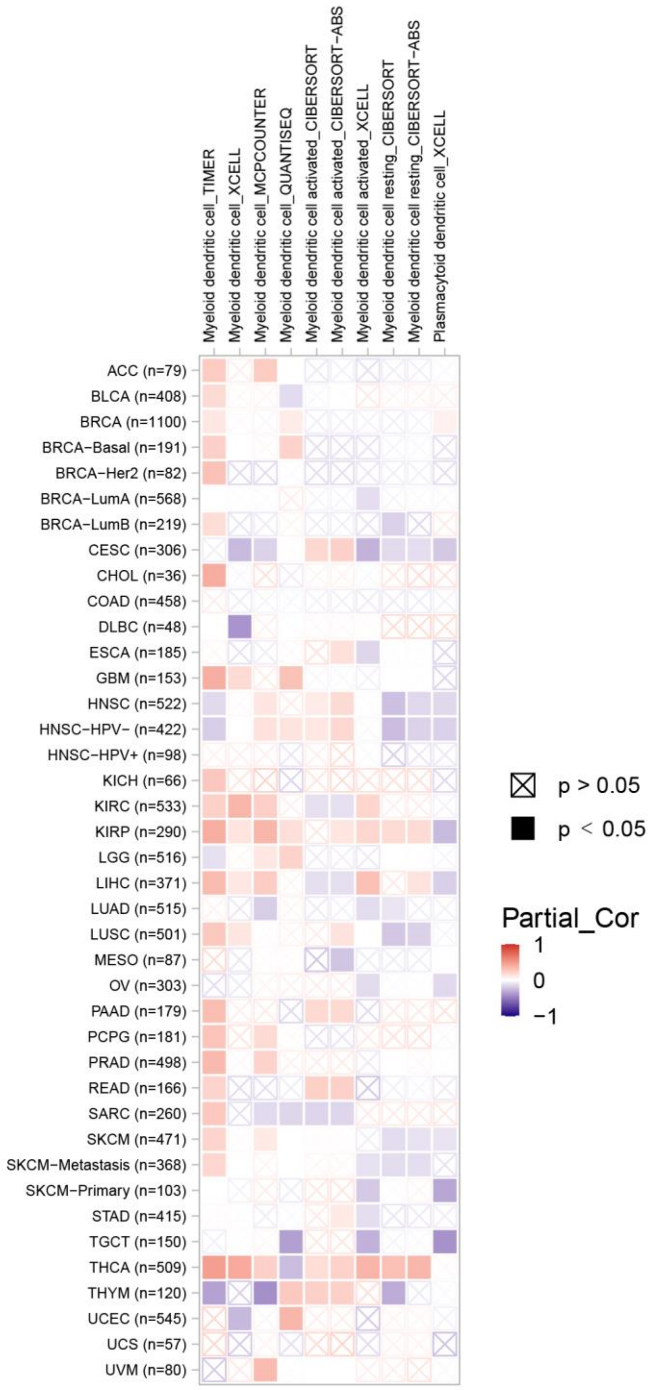

FIGURE S8 The Correlations of *CEMIP* expression with dendritic cell infiltration level across various types of cancers analyzed by all algorithms provided in TIMER2.0 web. The red squares represent positive correlations, while the blue squares represent negative correlations with statistical significance ($p < 0.05$).

TABLE S1 The information and sample size of TCGA BC datasets

Characteristics	Living(N=683)	Deceased(N=106)	Total(N=788)	p-value
Stage				2.00E-08
I	123(15.71%)	15(1.92%)	138(17.62%)	
II	418(53.38%)	46(5.87%)	464(59.26%)	
III	139(17.75%)	34(4.34%)	173(22.09%)	
IV	2(0.26%)	6(0.77%)	8(1.02%)	
Age				
Mean ± SD	57.07±12.68	60.97±14.75	57.59±13.04	
Median [min, max]	56.00[27.00,90.00]	61.00[26.00,90.00]	57.00[26.00,90.00]	
ER				0.48
Negative	151(20.30%)	27(3.63%)	178(23.92%)	
Positive	494(66.40%)	72(9.68%)	566(76.08%)	
HER2				0.78
Negative	537(71.60%)	79(10.53%)	616(82.13%)	
Positive	115(15.33%)	19(2.53%)	134(17.87%)	
PR				0.82
Negative	210(28.26%)	34(4.58%)	244(32.84%)	
Positive	434(58.41%)	65(8.75%)	499(67.16%)	
Subtypes				0.53
Normal-like	14(2.34%)	5(0.83%)	19(3.17%)	
Luminal A	261(43.57%)	42(7.01%)	303(50.58%)	
Luminal B	105(17.53%)	22(3.67%)	127(21.20%)	
HER2+	35(5.84%)	8(1.34%)	43(7.18%)	
Basal-like	92(15.36%)	15(2.50%)	107(17.86%)	
Tumor				4.70E-06
T1	185(23.51%)	27(3.43%)	212(26.94%)	
T2	410(52.10%)	53(6.73%)	463(58.83%)	
T3	76(9.66%)	14(1.78%)	90(11.44%)	
T4	11(1.40%)	11(1.40%)	22(2.80%)	
Node				0.01
N0	346(44.13%)	36(4.59%)	382(48.72%)	
N1	221(28.19%)	45(5.74%)	266(33.93%)	
N2	73(9.31%)	18(2.30%)	91(11.61%)	
N3	39(4.97%)	6(0.77%)	45(5.74%)	
Metastasis				3.20E-08
Negative	593(85.45%)	91(13.11%)	684(98.56%)	
Positive	2(0.29%)	8(1.15%)	10(1.44%)	
TP53				0.37
Non-mutated	459(58.17%)	66(8.37%)	525(66.54%)	
Mutated	224(28.39%)	40(5.07%)	264(33.46%)	

TABLE S2 Univariate and multivariate OS analysis using Cox Proportional Hazards model carried out by R with TCGA BC datasets

Variables	Univariate analysis		Multivariate analysis	
	HR (95%CI)	P-value	HR (95%CI)	P-value
Stage	2.2 (1.6-2.9)	2.3e-07***	1.92 (0.86-4.29)	0.113
Age	1 (1-1.1)	3.3e-06***	1.05 (1.03-1.07)	4.18e-07***
ER	0.88 (0.56-1.4)	0.59	0.82 (0.34-1.95)	0.647
HER2	1.3 (0.8-2.2)	0.27	1.41 (0.77-2.61)	0.266
PR	1 (0.66-1.5)	0.98	0.91 (0.41-2.04)	0.828
Subtypes	0.97 (0.82-1.1)	0.73	0.86 (0.63-1.18)	0.343
Tumor	1.5 (1.2-1.9)	8e-04***	1.01 (0.64-1.61)	0.957
Node	1.5 (1.2-1.9)	0.00015***	1.09 (0.68-1.76)	0.717
Metastasis	4 (1.8-8.5)	0.00045***	1.71 (0.50-5.79)	0.390
TP53	1.1 (0.76-1.7)	0.55	1.19 (0.63-2.26)	0.589
CEMIP	1.1 (1-1.2)	0.042*	1.17 (1.02-1.34)	0.028*

*p<0.05, **p<0.01, ***p<0.001. HR, hazard ratio; CI, confidence interval.

TABLE S3 Ten Differentially expressed predicted upstream TFs and 26 interacted kinases of *CEMIP*, validation using UALCAN web based on TCGA and expression correlations of *CEMIP* with validated TFs using bc-GenExMiner

Gene symbol	Log2FC	adj.P.Val	Direction	r	p-value
KLF5	2.04	0.000000282	down***		
TRIM28	1.59	8.60E-14	up***	-0.09***	< 0.0001
KDM2B	1.35	3.82E-12	up***		
EZH2	4.22	2.23E-29	up***	0.07***	< 0.0001
EGR1	-1.76	7.57E-09	down***	-0.03**	0.0017
ZNF217	1.44	2.22E-10	up***		
TFAP2C	2.06	1.00E-14	down***		
CTBP2	1.11	3.12E-13	up***	-0.07***	< 0.0001
JUN	-1.50	2.40E-13	down***	-0.04***	0.0004
ZMYND8	1.21	5.43E-14	up***		
TRIB3	1.135	1.24E-10	up***		
MAPKAPK2	1.48	1.02E-27	up***		
PTK2	1.22	1.04E-11	up***		
FYN	-1.09	1.29E-06	down***		
MELK	3.05	3.55E-15	up***		
TESK1	-1.30	3.24E-14	down**		
CDK18	1.38	1.46E-17	down***		
NTRK2	-1.05	6.81E-08	down***		
TYK2	1.06	7.45E-13	up***		
PRKD2	1.36	1.76E-23	up***		
MARK2	1.43	2.54E-29	up***		
PDK3	1.69	7.07E-12	up***		
PIM2	1.71	5.97E-20	up***		
MAP3K9	2.07	2.01E-38	up**		
ERBB4	1.58	2.51E-07	down ns		
PAK6	1.64	5.95E-15	up***		
RET	1.21	3.81E-05	up***		
TYRO3	-1.10	1.66E-31	down***		
PLK2	1.35	0.000202427	down*		
PIK3CA	-1.25	1.09E-18	down***		
ERBB3	1.65	2.31E-19	up***		
TGFBR2	-1.03	1.31E-08	down***		
DDR2	-2.64	1.80E-29	down***		
PTK6	2.18	1.03E-24	up***		
ADRBK2(GRK3)	-1.70	3.33E-18	down***		

*p<0.05, **p<0.01, ***p<0.001. ns, no significance. adj.P.Val, adjusted p value.

TABLE S4 Binding sites of *EGR1* and *JUN* in *CEMIP* promoter region predicted by JASPAR[2022] database

Matrix ID	Name	Score	Relative score	Sequence ID	Start	End	Strand	Predicted sequence
**12389**	MA0162.4.EGR1	13.441443	0.8948024953568379	NC_000015.10:80777370-80779469	1867	1880	-	ACGCGCCCCCGCCT
**12389**	MA0162.4.EGR1	12.419216	0.8769261891288744	NC_000015.10:80777370-80779469	1923	1936	-	TGACGCCCGCGCGG
**12389**	MA0162.4.EGR1	9.671872	0.828881724289698	NC_000015.10:80777370-80779469	1900	1913	-	TCCCTCCCTCGCCC
**12389**	MA0162.4.EGR1	9.6671915	0.8287998712227431	NC_000015.10:80777370-80779469	1869	1882	-	CCACGCGCCCCCGC
**12389**	MA0162.4.EGR1	9.591991	0.8274848019707878	NC_000015.10:80777370-80779469	1636	1649	-	CCCCGCTCGCGCCC
**12389**	MA0162.4.EGR1	8.499983	0.8083881879265736	NC_000015.10:80777370-80779469	1843	1856	+	CAGGGCCCCCGCCC
**10609**	MA0489.1.JUN	15.064485	0.9587863334589656	NC_000015.10:80777370-80779469	897	910	-	AGAGTGTGACTCAG
**10609**	MA0489.1.JUN	13.017211	0.9324417850358804	NC_000015.10:80777370-80779469	892	905	+	GGGGGCTGAGTCAC
**10609**	MA0489.1.JUN	6.926435	0.8540649918332043	NC_000015.10:80777370-80779469	1947	1960	+	GGGGGAGGAGTCAG
**10609**	MA0489.1.JUN	5.0221696	0.829560689496377	NC_000015.10:80777370-80779469	329	342	+	CCAGGCTGATTCAA
**10609**	MA0489.1.JUN	4.218792	0.8192227354189613	NC_000015.10:80777370-80779469	675	688	+	ACCAGGTGACAAAG

TABLE S5 Annotation terms *CEMIP* involved in from enrichment analysis of validated kinases interacted with *CEMIP* performed by Metascape web.

GO	Category	Description	LogP	Enrichment	Gene symbol
GO:0001934	GO Biological Processes	positive regulation of protein phosphorylation	-16.43	23.42	MARK2|ERBB3|FYN|NTRK2|DDR2|PIK3CA|PTK2|PTK6|RET|TESK1|TGFBR2|PRKD2|PAK6|CEMIP
GO:0043549	GO Biological Processes	regulation of kinase activity	-16.17	22.44	MARK2|ERBB3|NTRK2|DDR2|PIK3CA|PTK2|PTK6|RET|TESK1|TGFBR2|TYRO3|PAK6|CEMIP|TRIB3
GO:0051347	GO Biological Processes	positive regulation of transferase activity	-14.40	26.10	MARK2|ERBB3|NTRK2|DDR2|PIK3CA|PTK2|RET|TGFBR2|TYRO3|PAK6|CEMIP|TRIB3
GO:0033674	GO Biological Processes	positive regulation of kinase activity	-13.45	28.06	MARK2|ERBB3|NTRK2|DDR2|PIK3CA|PTK2|RET|TGFBR2|TYRO3|PAK6|CEMIP
GO:0045859	GO Biological Processes	regulation of protein kinase activity	-12.05	20.88	MARK2|ERBB3|DDR2|PIK3CA|PTK2|PTK6|TESK1|TGFBR2|PAK6|CEMIP|TRIB3
GO:0045860	GO Biological Processes	positive regulation of protein kinase activity	-9.21	24.55	MARK2|ERBB3|DDR2|PIK3CA|PTK2|TGFBR2|PAK6|CEMIP
GO:0018107	GO Biological Processes	peptidyl-threonine phosphorylation	-5.63	41.86	MARK2|TGFBR2|PRKD2|CEMIP
GO:0018210	GO Biological Processes	peptidyl-threonine modification	-5.50	38.87	MARK2|TGFBR2|PRKD2|CEMIP
GO:0030335	GO Biological Processes	positive regulation of cell migration	-5.36	13.21	DDR2|PTK2|RET|TGFBR2|PRKD2|CEMIP
GO:2000147	GO Biological Processes	positive regulation of cell motility	-5.25	12.62	DDR2|PTK2|RET|TGFBR2|PRKD2|CEMIP
GO:0051272	GO Biological Processes	positive regulation of cellular component movement	-5.19	12.37	DDR2|PTK2|RET|TGFBR2|PRKD2|CEMIP
GO:0040017	GO Biological Processes	positive regulation of locomotion	-5.19	12.33	DDR2|PTK2|RET|TGFBR2|PRKD2|CEMIP
GO:1903829	GO Biological Processes	positive regulation of cellular protein localization	-2.85	13.31	FYN|TESK1|CEMIP
GO:0071900	GO Biological Processes	regulation of protein serine/threonine kinase activity	-2.50	10.04	TESK1|CEMIP|TRIB3
GO:1903827	GO Biological Processes	regulation of cellular protein localization	-2.06	6.97	FYN|TESK1|CEMIP

TABLE S6 Expression level of potential kinases interacted with *CEMIP* participating in cell migration analyzed based on GSE42568 dataset

Gene symbol	Log2FC (BC vs normal)	adj.P.Val
DDR2	-2.638146086	1.80E-29
PTK2	1.224672223	1.04E-11
RET	1.206934303	3.81E-05
TGFBR2	-1.028307603	1.31E-08
PRKD2	1.358350129	1.76E-23

TABLE S7 Gene Set Enrichment Analysis of *CEMIP* based on GSE42568 dataset and gene sets.

KEGG pathways	SIZE	NES	NOM p-val
Positively correlated pathways			
1. KEGG_ANTIGEN_PROCESSING_AND_PRESENTATION	74	1.78	0.011
2. KEGG_GLYCOSAMINOGLYCAN_BIOSYNTHESIS_CHONDROITIN_SULFATE	21	1.67	0.016
3. KEGG_ASTHMA	26	1.65	0.004
4. KEGG_AUTOIMMUNE_THYROID_DISEASE	46	1.61	0.044
5. KEGG_FC_EPSILON_RI_SIGNALING_PATHWAY	73	1.61	0.040
6. KEGG_STEROID_BIOSYNTHESIS	16	1.58	0.023
7. KEGG_TYPE_I_DIABETES_MELLITUS	39	1.57	0.038
8. KEGG_LEISHMANIA_INFECTION	67	1.57	0.038
9. KEGG_GLYCOSAMINOGLYCAN_BIOSYNTHESIS_HEPARAN_SULFATE	26	1.46	0.037
10. KEGG_BLADDER_CANCER	41	1.42	0.049
HALLMARKs			
1. HALLMARK_ALLOGRAFT_REJECTION	193	1.56	0.042
2. HALLMARK_EPITHELIAL_MESENCHYMAL_TRANSITION	195	1.48	0.048
